# PRDM16 modulates aspects of cell cycle dynamics and maturation in human iPSC-derived cardiomyocytes

**DOI:** 10.1016/j.stemcr.2026.103005

**Published:** 2026-07-09

**Authors:** Kanae Tani, Yasuko Matsumura, Misato Nishikawa, Megumi Narita, Amanda Putri Elvandari, Antonio Lucena-Cacace, Yoshinori Yoshida

**Affiliations:** 1 Center for iPS Cell Research and Application, Kyoto University, Kyoto, Japan

**Keywords:** hiPSC-CMs, PRDM16, CDK1, phospho-AKT, TNNI3, EHT, cell-cycle regulation, sarcomere maturation, mitochondrial metabolism, cardiomyocyte maturation

## Abstract

Deciphering how cardiomyocytes exit proliferation and acquire postnatal maturity is essential for understanding heart development and regenerative potential. We identify PRDM16 as a regulator that influences the balance between proliferation and maturation in human iPSC-derived cardiomyocytes (hiPSC-CMs). Mechanistically, PRDM16 controls proliferative activity by modulating CDK1 and phospho-AKT, two central drivers of cardiomyocyte cell cycle progression. Loss of PRDM16 profoundly disrupts metabolic and structural maturation, yielding abnormal sarcomeric protein ratios and impaired engineered heart tissue function. In contrast, moderate PRDM16 overexpression promotes metabolic rewiring and structural remodeling characteristic of mature cardiomyocytes. These findings support a model in which PRDM16 functions as a context-dependent modulator governing the transition from proliferation to maturation. By demonstrating that transient downregulation favors proliferation while sustained expression drives maturation, our work highlights PRDM16 as both a potential developmental regulator and a therapeutic target for enhancing cardiomyocyte regeneration, cardiac repair, and the functional engineering of human heart tissues.

## Highlights

• PRDM16 regulates the proliferation-maturation balance in hiPSC-derived cardiomyocytes
• PRDM16 suppression sustains proliferative competence via CDK1 and phospho-AKT
• PRDM16 overexpression promotes hypertrophy and postnatal-like sarcomeric maturation
• Loss of PRDM16 impairs metabolic, structural, and functional maturation

## Graphical abstract

**Figure:**
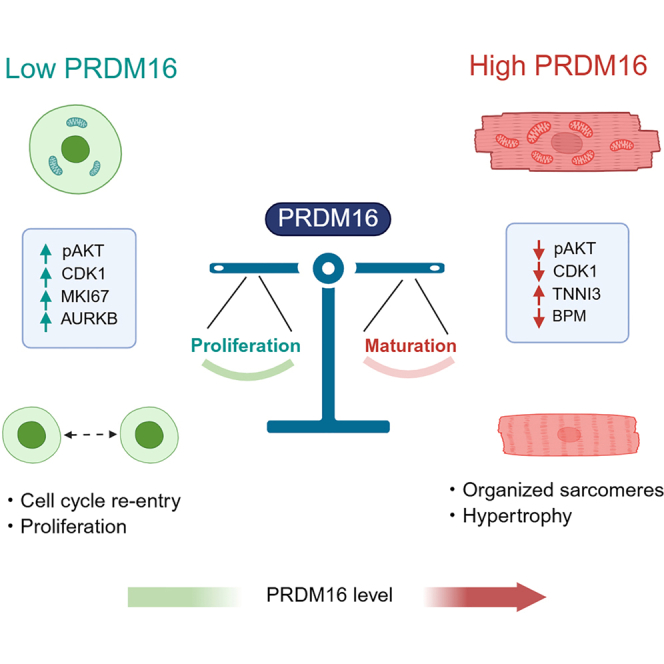

## Teaser

Tani et al. identify PRDM16 as a regulator of the proliferation-maturation switch in human iPSC-derived cardiomyocytes. PRDM16 silencing sustains proliferative potential through the phospho-AKT-CDK1 axis, while impairing mitochondrial and sarcomeric maturation. Conversely, PRDM16 overexpression suppresses proliferation and promotes postnatal-like energetic and structural maturation. These findings position PRDM16 as a key determinant of cardiomyocyte functional competence.

## Introduction

Cardiovascular disease remains the leading global cause of mortality (Satoh et al., 2025), and the limited regenerative capacity (Iismaa et al., 2018) of the adult human heart magnifies the impact of every lost cardiomyocyte. Human induced-pluripotent stem cell-derived cardiomyocytes (hiPSC-CMs) offer a renewable, patient-specific platform for modeling disease, screening drugs, and developing engineered heart tissues (EHTs) (Cerneckis et al., 2024; Goldfracht et al., 2019; Karakikes et al., 2015; Paik et al., 2020; Tani and Tohyama, 2022; Tian et al., 2022; Clancy and Santana, 2024). hiPSC-CMs remain fetal-like, proliferative, structurally immature, glycolytic, and limited for therapy. Deciphering how proliferation is silenced and maturation induced is a key challenge in cardiac biology. During murine heart development, the transcriptional regulator Prdm16 is highly enriched in the compact myocardium and is required to preserve ventricular identity and constrain hyper-proliferation (Wu et al., 2022). Human PRDM16 (PR domain-containing 16) is a zinc-finger-containing transcriptional regulator belonging to the PRDM family, characterized by its N-terminal PR domain and multiple C2H2 zinc fingers. PRDM16 functions as both a transcriptional activator and repressor depending on its interacting partners, orchestrating cell fate decisions across diverse lineages, including the nervous system, adipose tissue, and the cardiovascular system (Aguilo et al., 2011; Gudmundsson et al., 2020; He et al., 2025; Thompson et al., 2023; Van Wauwe et al., 2025; Wang et al., 2023; Zhao et al., 2023). Its ability to recruit chromatin-modifying complexes such as histone methyltransferases and corepressors underlies its broad impact on gene expression and cellular identity (Biferali et al., 2021). Loss of Prdm16 in mice leads to ventricular non-compaction and heart failure (Sun et al., 2023; Wu et al., 2022), implicating Prdm16 as a gatekeeper of the proliferative-to-quiescent transition that precedes postnatal hypertrophic growth. However, whether PRDM16 plays a key role in human cardiomyocyte maturation, and how it interfaces with the canonical cell-cycle machinery, remains unknown.

Our analysis in hiPSC-CMs carrying a fluorescent ubiquitination-based cell cycle indicator (FUCCI) cell-cycle reporter (Kasamoto et al., 2023) revealed that *PRDM16* expression oscillates with the cell cycle, falling in S/G_2_/M and rising in quiescent G_0_/G_1_ cells. Concomitantly, PRDM16-downregulating subsets exhibit elevated *CDK1*, *CDK4*, *PLK1*, and *PCNA*, underscoring a potential repressive function on the core cyclin-dependent kinase (CDK) axis. Conversely, PRDM16-positive cells upregulate adult sarcomere genes such as *MYH7*, *MYL2*, and *TNNI3*, hinting at a coordinated switch from proliferation to maturation. Together, these correlative observations prompt further investigation into whether PRDM16 is sufficient to restrain the CDK network and enforce quiescence in human cardiomyocytes, whether its downregulation mechanistically limits sarcomeric and metabolic maturation, and whether transient modulation of PRDM16 levels can be leveraged to optimize the balance between proliferation and functional maturation to enhance EHT performance.

We combine genetics, flow cytometry, and tissue engineering to define PRDM16’s role in hiPSC-CMs. Acute PRDM16 knockdown boosts CDK1, overrides mTOR-p53-mediated quiescence, and re-activates the cell proliferation axis, while controlled modulation of PRDM16 promotes maturation and suppresses phospho-AKT/CDK1. Our findings demonstrate that PRDM16 acts as a dual regulator that couples proliferation restraint with structural maturation. Thus, PRDM16 links developmental timing to the CDK engine, offering a molecular handle to balance growth and maturation for cardiac repair and precision hiPSC-CM models.

## Results

### PRDM16 expression varies with cell-cycle dynamics and maturation in hiPSC-CMs

**Figure 1:**
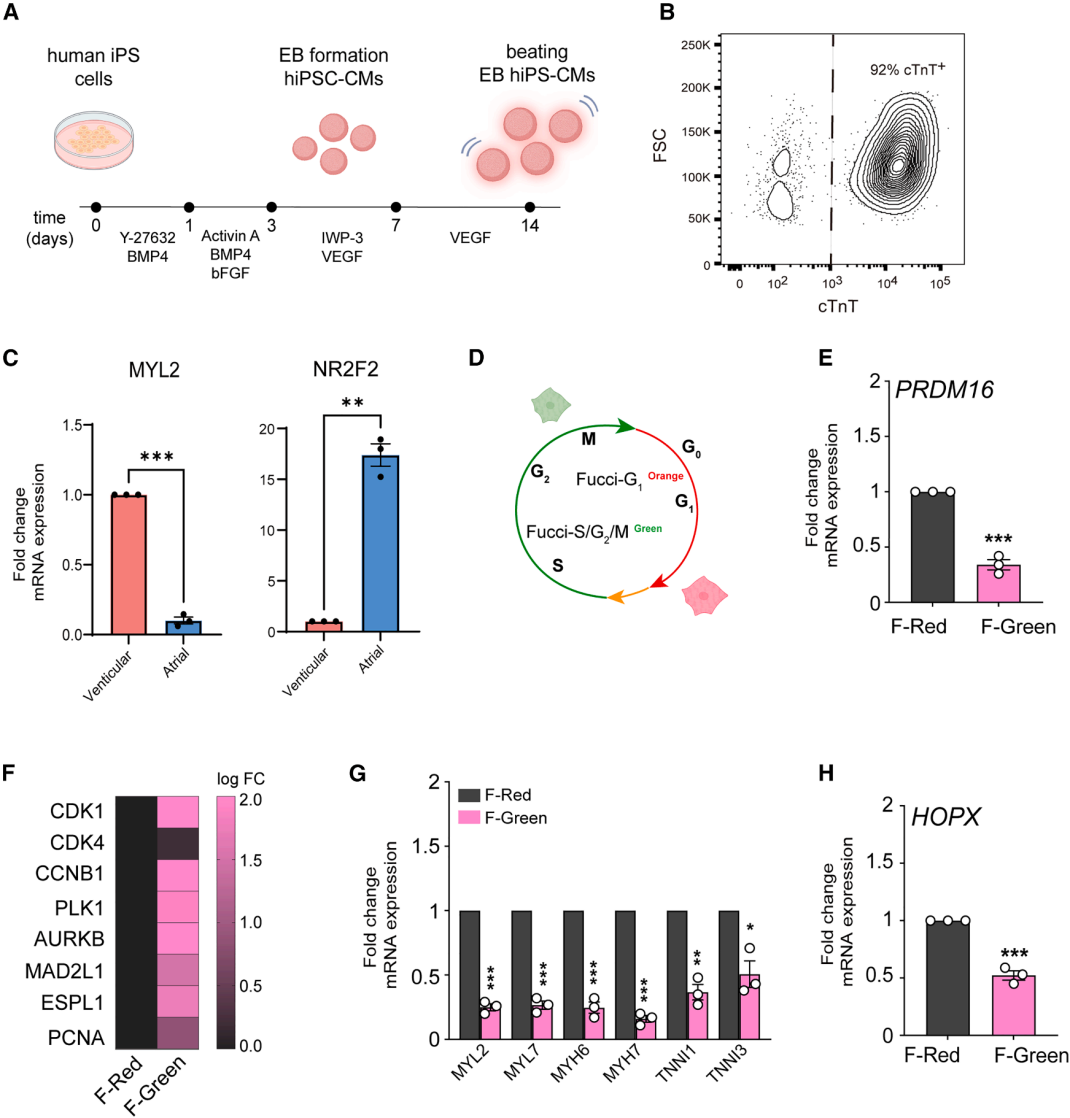
Establishment of ventricular cardiomyocyte identity and FUCCI-based analysis reveal cell-cycle-coupled PRDM16 expression in hiPSC-CMs (A) Schematic of ventricular cardiomyocytes differentiation from day 0 to day 14. (B) Flow cytometry plot showing enrichment of cTNT^+^ cardiomyocytes following sorting, with representative forward scatter (FSC) versus cardiac troponin T (cTNT) gating. (C) Quantitative RT-PCR analysis of ventricular (*MYL2*) and atrial (*NR2F2*) marker expression in ventricular versus atrial hiPSC-derived cardiomyocytes. Data are shown as mean ± SEM; ^∗∗^*p* < 0.01, ^∗∗∗^*p* < 0.001, *n* = 3 independent cultures per condition. (D) Diagram of the FUCCI cell-cycle reporter system used to identify G_1_ (Fucci-G_1_, orange) and S/G_2_/M (Fucci-S/G_2_/M, green) phases in live cardiomyocytes. (E) Relative *PRDM16* mRNA expression in FUCCI-sorted hiPSC-derived cardiomyocytes in G_1_ (F-red) and S/G_2_/M (F-green) phases. Data are shown as mean ± SD; ^∗∗∗^*p* < 0.001, *n* = 3 independent cultures per condition. (F) Heatmap showing log fold change of cell-cycle-related genes in F-red versus F-green cardiomyocytes, indicating enrichment of proliferative markers in F-green cells. (G) Relative mRNA expression of sarcomeric and isoform markers (*MYL2*, *MYL7*, *MYH6*, *MYH7*, *TNNI1*, and *TNNI3*) in FUCCI-sorted F-red and F-green cardiomyocytes. Data are shown as mean ± SD; ^∗^*p* < 0.05, ^∗∗^*p* < 0.01, ^∗∗∗^*p* < 0.001, *n* = 3 independent cultures per condition. (H) Relative *HOPX* mRNA expression in F-red and F-green cardiomyocytes, showing reduced expression in cycling cells, *n* = 3 independent cultures per condition.

Prdm16 has been identified as a transcription factor enriched in the compact myocardium and is essential for maintaining compact cardiomyocyte identity in the left ventricle of the developing murine heart (Wu et al., 2022). To investigate whether PRDM16 plays a similar regulatory role in human cardiomyocytes, we first established a high-purity population of hiPSC-CMs (Figures 1A and 1B). These cells exhibited a distinct ventricular phenotype, characterized by enrichment of ventricular markers (*MYL2*, *IRX4*, and *HEY2*) and depletion of atrial markers (*NR2F2*, *NPPA*, and *PITX2*) compared with atrial-induced cardiomyocytes (Figures 1C and S1A), as well as protein expression of MYL2 (Figure S1B).

To examine the relationship between PRDM16 and cardiomyocyte cell-cycle progression, we analyzed its expression in proliferative versus non-proliferative hiPSC-CMs using a dataset from FUCCI-reporter hiPSC-CMs (Kasamoto et al., 2023) (Figure 1D). PRDM16 expression was significantly reduced in green (S/G_2_/M) cells and increased in red (G_1_/G_0_) cardiomyocytes (Figure 1E), indicating dynamic regulation across the cardiomyocyte cell cycle.

Consistent with this pattern, FUCCI-green (F-green) cells exhibited lower *PRDM16* levels alongside elevated expression of cell-cycle-associated genes, including *CDK1*, *CDK4*, *PLK1*, and *PCNA* (Figure 1F), confirming their proliferative state.

We next explored the relationship between *PRDM16* expression and cardiomyocyte maturation. Analysis of sarcomere-related transcripts revealed that proliferative F-green cells displayed marked downregulation of sarcomere-related markers, including *MYL2*, *MYH7*, and *TNNI3* (Figure 1G). In addition, *PRDM16*-low F-green cells expressed approximately half the level of *HOPX* compared with quiescent FUCCI-red (F-red) cells (Figure 1H). Together, these findings indicate that *PRDM16* expression is associated with cell-cycle exit and increased maturation in hiPSC-CMs.

### Human PRDM16 mediates a proliferative gateway in hiPSC-CMs by promoting quiescence through regulation of CDK1 and phospho-AKT

**Figure 2:**
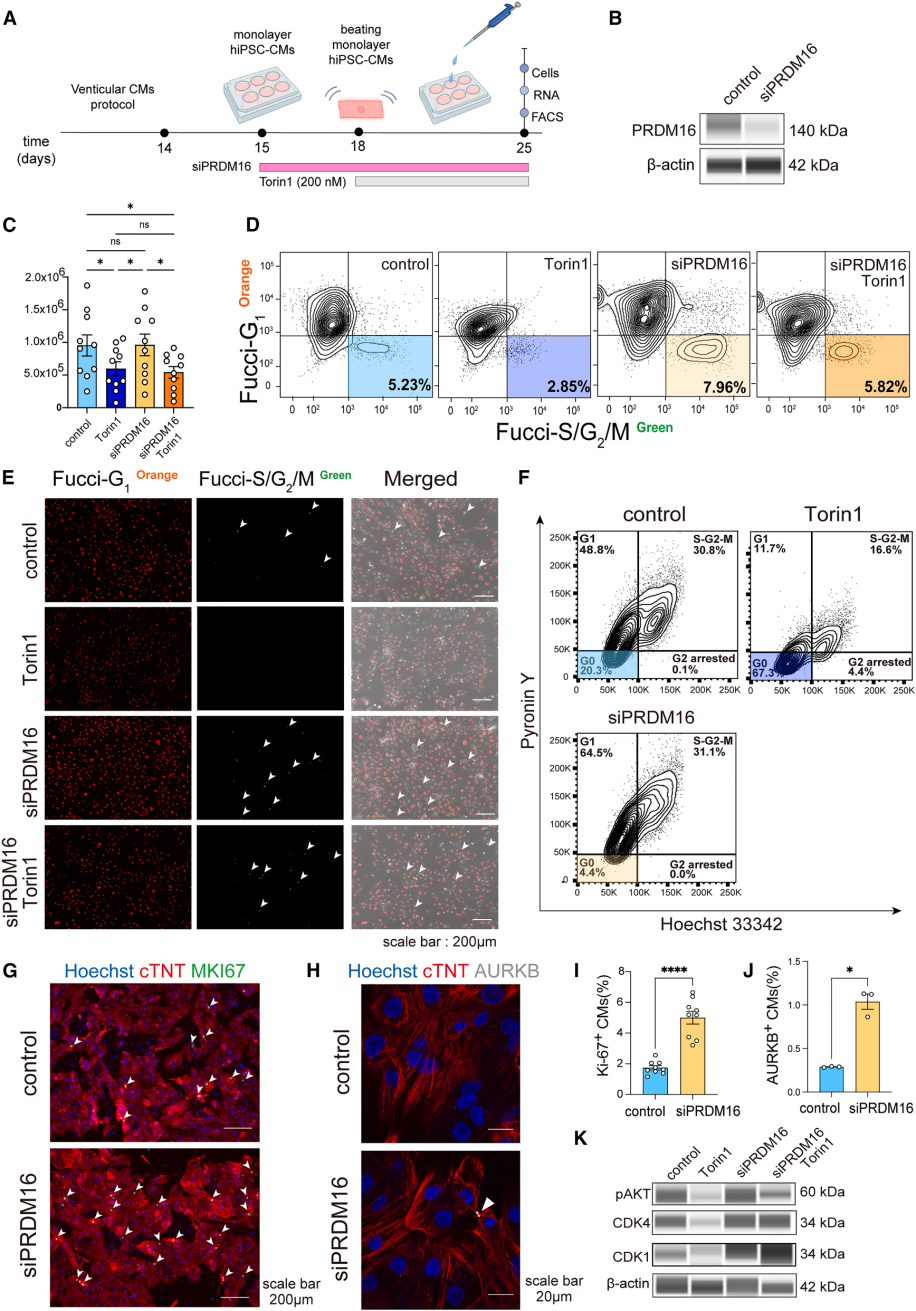
PRDM16 is required for AKT-dependent cell-cycle progression in human iPSC-derived cardiomyocytes (A) Schematic of experimental timeline used to generate beating monolayer hiPSC-derived cardiomyocytes, followed by siPRDM16 knockdown and mTOR inhibition (Torin1, 200 nM). Time points for cell collection for imaging, RNA extraction and flow cytometry (FACS) are indicated. (B) Immunoblot confirming PRDM16 knockdown efficiency in hiPSC-CMs treated with control or siPRDM16. β-actin serves as a loading control. (C) Quantification of cell number across treatment conditions shown in Figure S2E. Data are shown as mean ± SEM. Statistical significance is indicated (*n* = 10 independent experiments; repeated measures one-way ANOVA, ^∗^*p* < 0.05; ns, not significant). (D) Flow cytometric quantification of FUCCI reporters showing the proportion of cells in S/G_2_/M phase under each experimental condition, highlighting increased cycling upon PRDM16 depletion and partial rescue by Torin1. (E) Representative fluorescence images of FUCCI-labeled hiPSC-CMs showing G_1_ (orange), S/G_2_/M (green), and merged signals under control, Torin1, siPRDM16, and siPRDM16 + Torin1 conditions. Arrowheads indicate cells in S/G_2_/M phases. (F) Flow cytometric analysis of DNA content (Hoechst 33342) and RNA content (Pyronin Y) to assess cell-cycle-phase distribution in control, Torin1-treated, and siPRDM16 cardiomyocytes. Percentages of cells in G_0_/G_1_, S/G_2_/M, and G_2_-arrested states are indicated. (G) Immunofluorescence staining for MKI67 (green) and TNNT2 (red) in control and siPRDM16 hiPSC-CMs, with Hoechst nuclear counterstain (blue). Arrowheads mark MKI67^+^ cardiomyocytes. Scale bars, 200μm. (H) Immunofluorescence staining for AURKB (gray) and TNNT2 (red) in control and siPRDM16 hiPSC-CMs, with Hoechst nuclear counterstain (blue). Arrowheads indicate AURKB^+^ cycling cardiomyocytes. Scale bars, 20μm. (I) Quantification of MKI67 positive cardiomyocytes shown in (G). Data are shown as mean ± SEM. Statistical significance is indicated (*n* = 6 independent experiments; Welch’s *t* test, ^∗∗∗∗^*p* < 0.0001.). (J) Quantification of AURKB positive cardiomyocytes shown in (H). Data are shown as mean ± SEM. Statistical significance is indicated (*n* = 3 independent experiments; Welch’s *t* test, ^∗^*p* < 0.05.). (K) Immunoblot analysis of phospho-AKT, CDK4, and CDK1 protein levels in control, Torin1-treated, siPRDM16, and combined siPRDM16 + Torin1 hiPSC-CMs. β-actin serves as a loading control. Uncropped images of these blots are shown in Figure S3A.

To establish a causal relationship between PRDM16 and maturation blockade via insufficient repression of the proliferative program, we differentiated ventricular hiPSC-CMs carrying the FUCCI reporter and transfected them with siRNA against *PRDM16* at the cardiomyocyte replating stage (day 15) (Figure 2A), using scrambled siRNA-transfected cells as “control”. We first confirmed the protein expression of PRDM16 following transfection (Figure 2B). Three days post-transfection, we treated the cells with Torin1, a dual mTORC inhibitor known to promote hiPSC-CM maturation through p53-mediated quiescence (Garbern et al., 2020).

Day 25 of hiPSC-CMs was denoted as the experimental endpoint. Starting from an equal initial cell density before transfection and observing no cellular loss due to lipofectamine-induced stress (Figure S2A), as indicated by unchanged levels of phospho-H2AX and phospho-p38 and reduced levels of phospho-p53 (Figures S2B–S2D), we found that Torin1 effectively arrested the residual proliferative capacity of hiPSC-CMs, whereas siPRDM16-expressing cells yielded a mildly higher number of cardiomyocytes at the endpoint (Figures 2C and S2E), suggesting that reduced PRDM16 levels unlock proliferative potential in hiPSC-CMs.

To correlate the quiescent state with impaired cell cycle progression, we analyzed FUCCI reporter states in siPRDM16-treated hiPSC-CMs. Quantitatively, we observed that Torin1 reduced the proportion of F-green (proliferative) cells from 5.23% to 2.85%. In contrast, PRDM16 knockdown increased the proportion of F-green cells to 7.96%, and this elevation persisted even under Torin1 treatment, which failed to reduce F-green cells to baseline levels seen in untransfected cells. (Figure 2D) Also, we observed a significant increase in F-green-positive nuclei per field, indicating that reduced PRDM16 expression permits hiPSC-CMs to re-enter the cell cycle and undergo nuclear and mitotic division (Figure 2E). This confirms that the proliferative drive conferred by PRDM16 downregulation overrides the quiescence-inducing effects of mTOR inhibition and p53 activation. To quantify PRDM16-induced quiescence, we performed fluorescence-activated cell sorting (FACS) analysis using Pyronin Y and Hoechst staining to assess G_0_-phase exit. Torin1 increased the G_0_ population from 20.3% to 67.3%, whereas PRDM16 knockdown drastically reduced the quiescent fraction to 4.44%, indicating active escape from G_0_ (Figure 2F). To confirm whether these pro-cell-cycle indicators were aligned with a bona fide increase in proliferation, we performed immunocytochemistry (ICC) analysis to detect cardiomyocytes (TNNT2) expressing MKI67 (Figures 2G and 2I) and AURKB as a mitotic proxy (Figures 2H and 2J). Consistent with our FUCCI and quiescence analyses, cells downregulating PRDM16 proliferated to a greater extent than their control counterparts.

At the protein level, PRDM16 knockdown significantly increased CDK1, CDK4, and phospho-AKT, and Torin1 failed to suppress these proteins as it does in control cells, confirming that PRDM16 loss mechanistically favors a proliferative state over maturation (Figures 2K and S3A).

Collectively, these findings demonstrate that PRDM16 acts as a mechanistic repressor of the cell cycle during hiPSC-CM development, and its downregulation disrupts the balance between proliferation and maturation. This positions PRDM16 as a potential target for reactivating cell cycle activity in postnatal cardiac tissues.

### PRDM16 acts as a transcriptional repressor suppressing cell cycle progression checkpoints in hiPSC-CMs

**Figure 3:**
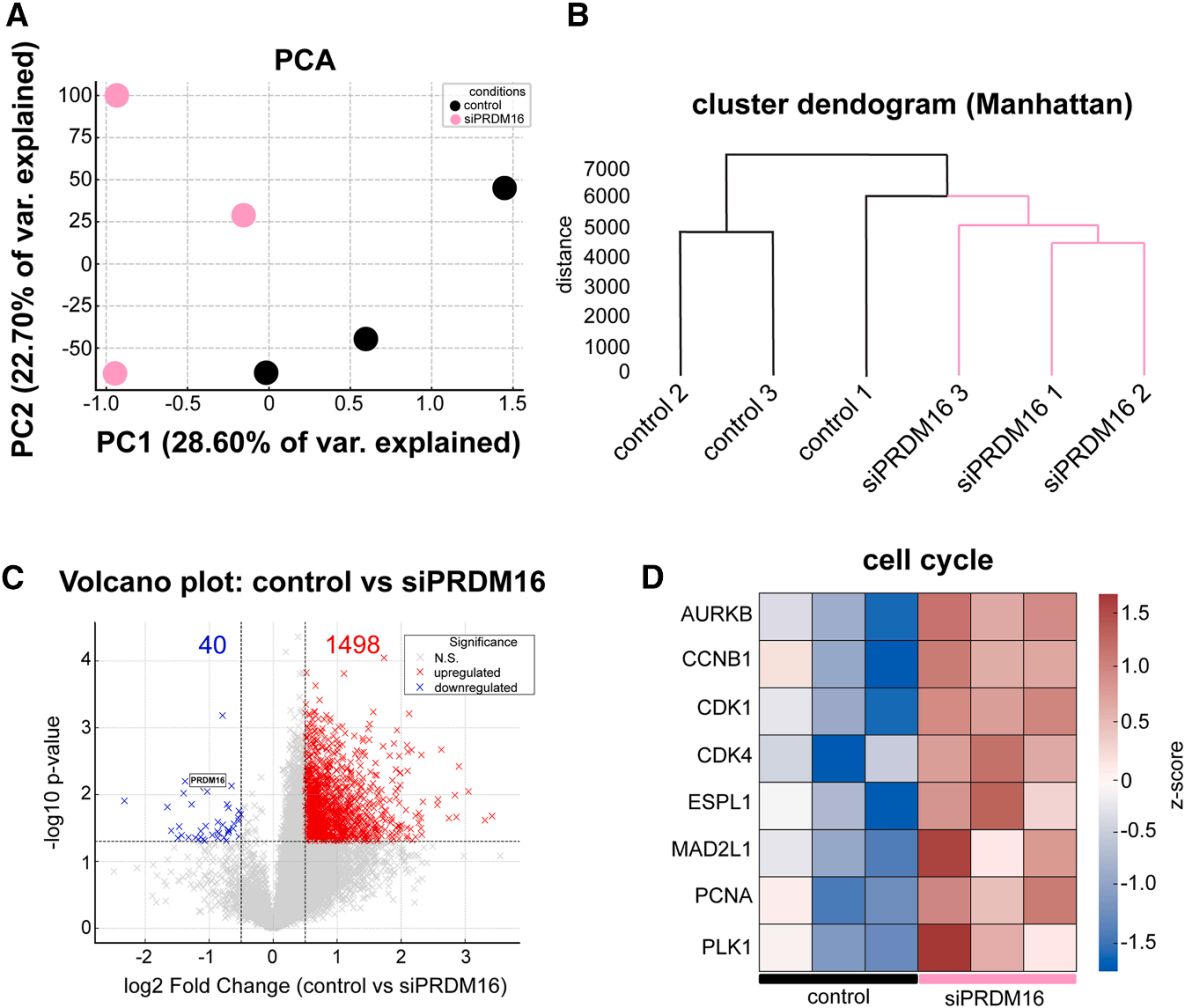
Transcriptomic profiling of PRDM16-deficient hiPSC-CMs reveals activation of cell cycle programs (A) Principal-component analysis (PCA) of bulk RNA-seq datasets from control and siPRDM16-transfected hiPSC-CMs, showing clear segregation between conditions (PC1: 28.6% variance, PC2: 22.7% variance). (B) Hierarchical clustering dendrogram (Manhattan distance) confirming distinct clustering of independent samples by condition. (C) Volcano plot of differentially expressed genes between control and siPRDM16 groups, highlighting upregulated (red, *n* = 1,498) and downregulated (blue, *n* = 40) transcripts, including enrichment of proliferation-associated factors. (D) Heatmap of selected cell cycle regulators (*AURKB*, *CCNB1*, *CDK1*, *CDK4*, *ESPL1*, *MAD2L1*, *PCNA*, and *PLK1*), demonstrating consistent upregulation in siPRDM16 hiPSC-CMs compared with controls.

To understand the transcriptomic perturbations caused by seven days of PRDM16 downregulation in developing hiPSC-CMs, we performed bulk RNA-seq on cardiomyocyte monolayers transfected with siRNA targeting *PRDM16* (siPRDM16) and compared them with cells transfected with a scrambled control sequence (scr). Principal-component analysis revealed that 28.60% of the variance was explained by PC1 (Figure 3A), with clear sample separation between the triplicates, highlighting the significant impact of PRDM16 on cellular transcriptomics. Hierarchical clustering further confirmed distinct clustering by condition in the dendrogram (Figure 3B), indicating a robust distribution of samples based on treatment.

Next, we performed a differential gene expression (DEG) analysis. The volcano plot (Figure 3C) revealed 1,498 significantly upregulated genes and only 40 downregulated genes, suggesting a marked asymmetry. This disproportionate number of upregulated genes indicates that PRDM16 primarily acts as a transcriptional repressor in hiPSC-CMs. Given that PRDM16 is expressed in a time-dependent manner during development, we hypothesized that its repression might target genes involved in cell cycle progression.

Indeed, several key cell cycle genes, including *CDK1*, *CDK4*, *PCNA*, and *PLK1*, were significantly upregulated following PRDM16 knockdown (Figure 3D), consistent with our previous observations using FUCCI-based cell cycle dynamics in wild-type cells.

Altogether, our data indicate that PRDM16 plays a global role in repressing proliferation in developing cardiomyocytes, supporting its function as an important regulator of cell cycling during cardiogenesis.

### Downregulation of PRDM16 hampers metabolic, structural, and functional maturation in hiPSC-CMs

Metabolic switching during developmental cardiogenesis is a critical reprogramming event in myocardial tissue, particularly postnatally. As cardiomyocytes mature, they transition to oxidative phosphorylation (OXPHOS) and fatty acid oxidation (FAO), accompanied by an increase in mitochondrial number. This metabolic shift coincides with proliferative exit and postmitotic arrest. In this context, we sought to understand how PRDM16 may impair or delay the metabolic maturation of cardiomyocytes.

**Figure 4:**
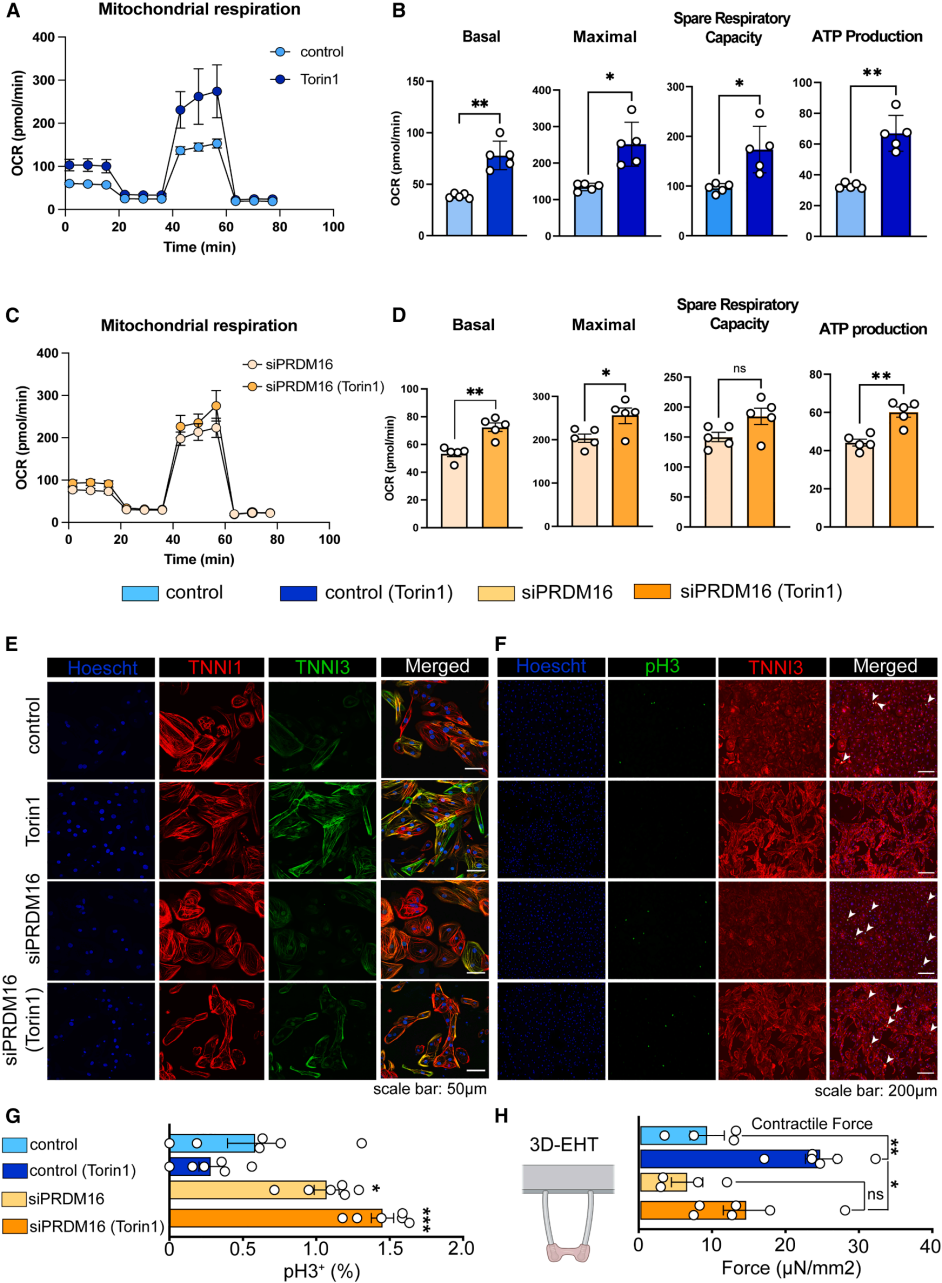
PRDM16 constrains metabolic maturation while limiting proliferative re-entry of human iPSC-derived cardiomyocytes (A and C) Seahorse XF analysis of mitochondrial respiration in hiPSC-derived cardiomyocytes under control and Torin1 treatment, siPRDM16 cardiomyocytes tested with or without Torin1. Oxygen consumption rate (OCR) is plotted over time following sequential injection of mitochondrial stressors. Data are shown as mean ± SD (*n* = 5 wells from a single representative differentiation experiment). (B and D) Quantification of basal respiration, maximal respiration, spare respiratory capacity, and ATP-linked respiration from Seahorse measurements shown in (A). Data are presented as mean ± SD (*n* = 5 wells from a single representative differentiation experiment); statistical significance was determined by Welch’s *t* test (^∗^*p* < 0.05, ^∗∗^*p* < 0.01, and “ns” denotes not significant). (E) Immunofluorescence staining of hiPSC-CMs for TNNI1 (red; immature isoform) and TNNI3 (green; mature isoform) under control, Torin1, siPRDM16, and siPRDM16 + Torin1 conditions. Nuclei are counterstained with Hoechst (blue). Scale bars, 50 μm. (F) Immunofluorescence staining for phospho-histone H3 (pH3) and TNNI3 to identify proliferating cardiomyocytes across experimental conditions. Arrowheads indicate pH3^+^ cardiomyocytes. Scale bars, 200 μm. (G) Quantification of pH3^+^ cardiomyocytes expressed as a percentage of total cells under each condition. (H) Measurement of contractile force using three-dimensional engineered heart tissues (3D-EHTs) generated from control, Torin1-treated, siPRDM16, and siPRDM16 + Torin1 hiPSC-CMs. Left, schematic of the 3D-EHT platform. Right, quantification of peak contractile force (μN/mm^2^). Data for (G) and (H) are shown as individual data points with mean ± SD; statistical significance was determined by one-way ANOVA followed by Tukey’s test (^∗^*p* < 0.05, ^∗∗^*p* < 0.01, ^∗∗∗^*p* < 0.001, *n* = 6 independent experiments).

To address this, we systematically analyzed the oxygen consumption rate (OCR) as a proxy for mitochondrial respiration using the Seahorse Mito Stress Test, which evaluates key parameters of mitochondrial function. siPRDM16-treated cells exhibited a mild increase in OCR compared with the untreated control, possibly reflecting an actively cycling population. mTOR-inhibiting conditions, known to promote cardiomyocyte maturation, were evaluated using Torin1 as a positive control. While Torin1 treatment significantly enhanced OCR in both control cells (Figures 4A and 4B) and siPRDM16 cells (Figures 4C and 4D), the magnitude of the increase was lower in siPRDM16 cells, suggesting that reduced PRDM16 acts as a partial brake on metabolic maturation (Figure 4A). However, since siPRDM16 induces partial cell-cycle re-entry and proliferating cardiomyocytes rely on higher basal mitochondrial ATP production, baseline mitochondrial respiration is increased in siPRDM16 cells compared with controls (Figure 4D; basal). Torin1 treatment leads to a modest increase in basal OCR but a significant maximal respiration profile, albeit to a lesser extent than Torin1-treated control cells, suggesting a proliferation-maturation conflict and resulting in a readout distinct from the maturation phenotype observed in control + Torin1 conditions.

Collectively, these results suggest that PRDM16 downregulation mildly impairs metabolic maturation and limits the ability of cardiomyocytes to improve mitochondrial function under mTOR-inhibiting conditions, possibly due to a proliferative-biased phenotype in PRDM16 downregulating conditions.

We then assessed mitochondrial content and spatial organization in hiPSC-CMs with PRDM16 knockdown. Interestingly, while PRDM16-downregulated cells transiently increased mitochondrial number, both mitochondrial abundance and spatial distribution were disrupted under mTOR-inhibiting conditions. This observation aligns with the impaired mitochondrial function revealed by the Mito Stress Test (Figures S3B and S3C). OXPHOS protein levels remained relatively stable, suggesting no perturbation of the mitochondrial machinery in siPRDM16 cells (Figure S3D).

Cardiomyocyte maturity is often inversely correlated with mitotic proliferation and cellular expansion. Since we established a causal relationship between low levels of PRDM16 and active cell cycling, we next sought to investigate how sarcomere maturation is affected by PRDM16 expression. Expression of adult isoforms of cardiac troponin proteins represents a critical maturation switch, supporting postnatal features such as contractile capacity and proper electrophysiology.

We first performed ICC analysis on cardiomyocytes with PRDM16 downregulation to assess TNNI3 expression—the adult isoform of cardiac troponin I. We observed that while Torin1 treatment effectively increases TNNI3 protein levels and promotes sarcomere alignment (reflected in a morphological shift from round to elongated rod-shaped cells), PRDM16-downregulated cells fail to express TNNI3 under both basal and Torin1-stimulated conditions. These cells retained a round, immature morphology (Figure 4E).

Next, we quantitatively assessed the mitotic index under reduced PRDM16 levels. To this end, we performed ICC analysis using an antibody against phosphorylated histone H3 (pH3), a widely used marker of mitotic cells (Figure 4F). siPRDM16-treated hiPSC-CMs consistently displayed a significant increase in pH3^+^ cells (approximately 2-fold), while Torin1 treatment failed to reduce this number, confirming that low PRDM16 levels maintain a permissive state for cell cycle re-entry and proliferation in hiPSC-CMs (Figure 4G), as we confirmed by observing increased MKI67 and AURKB protein levels (Figures 2G–2J).

Finally, to evaluate the functional contribution of PRDM16 in EHTs, we generated EHTs from both control and PRDM16-downregulated cardiomyocytes. Although no overt morphological differences were observed (Figure S4A), PRDM16-deficient EHTs failed to develop the contractile force seen in controls under mTOR-inhibiting conditions (Figure 4H). To examine whether this contractile failure was accompanied by altered sarcomeric isoform alteration, we analyzed the transcript levels of sarcomeric isoforms (*TNNI1*, *TNNI3*, *MYH6*, *MYH7*, and *MYL7*) by RNA-seq. PRDM16-deficient cardiomyocytes did not acquire the mature sarcomeric isoform profile, even following Torin1 treatment, consistent with impaired sarcomeric maturation (Figure S4B). Altogether, these findings demonstrate that PRDM16 is crucial for cardiomyocyte biology, initially by regulating cell cycle progression through CDK1 suppression and subsequently by promoting metabolic and sarcomeric maturation in response to developmental cues.

### PRDM16 expression defines a threshold that permits maturation of hiPSC-CMs and non-pathological cellular hypertrophy via CDK1 suppression

**Figure 5:**
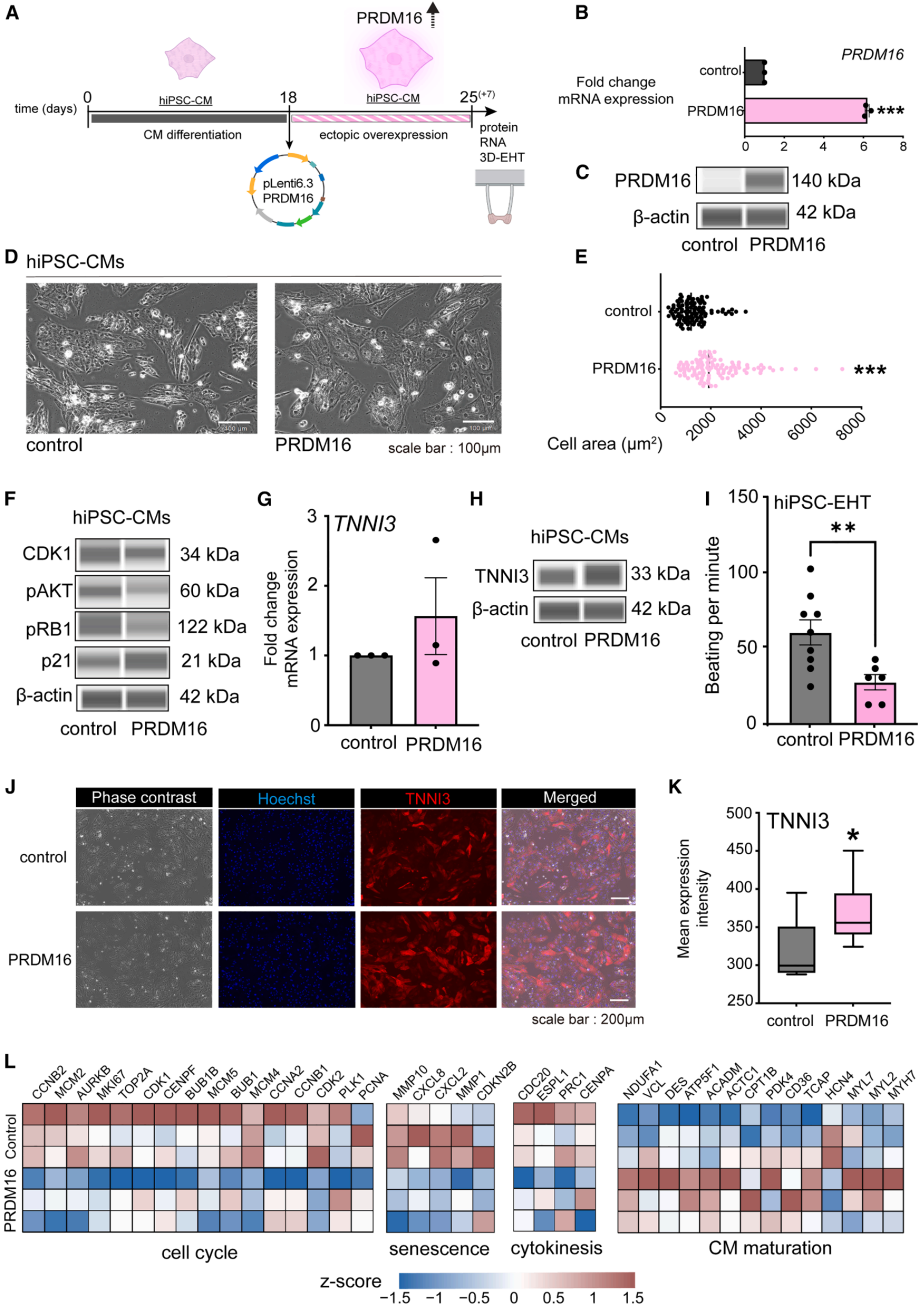
PRDM16 overexpression promotes cardiomyocyte maturation while suppressing cell cycle activity (A) Experimental schematic showing differentiation of hiPSCs into cardiomyocytes followed by ectopic overexpression of PRDM16 using a lentiviral vector (pLenti6.3-PRDM16). Cells were harvested for RNA, protein, imaging, and three-dimensional engineered heart tissue (3D-EHT) analysis at the indicated time points. (B) Quantitative RT-PCR analysis of *PRDM16* mRNA expression in control and PRDM16-overexpressing hiPSC-CMs. Statistical significance was determined by Welch’s *t* test. Data are shown as mean ± SEM; ^∗∗∗^*p* < 0.001, *n* = 3 independent experiments. (C) Immunoblot confirming PRDM16 protein overexpression in hiPSC-CMs. β-actin serves as a loading control. (D) Representative phase-contrast images of hiPSC-CM monolayers under control and PRDM16-overexpressing conditions. Scale bars, 100 μm. (E) Quantification of single-cell surface area in control and PRDM16-overexpressing hiPSC-CMs. Each dot represents one cell; statistical significance was determined by Welch’s *t* test, ^∗∗∗^*p* < 0.001, *n* = 100 cells per group. (F) Immunoblot analysis of cell-cycle and signaling regulators (CDK1, phospho-AKT, pRB1, and p21) in control and PRDM16-overexpressing hiPSC-CMs. β-actin serves as a loading control. Uncropped images of these blots are shown in Figure S6C. (G) Quantitative RT-PCR analysis of the maturation-associated sarcomeric gene *TNNI3* in control and PRDM16-overexpressing hiPSC-CMs. Data are presented as fold change relative to the control group. Error bars represent SEM, *n* = 3 independent experiments. (H) Immunoblot analysis of TNNI3 protein expression in control and PRDM16-overexpressing hiPSC-CMs. β-actin serves as a loading control. Uncropped images of these blots are shown in Figure S6C. (I) Beating frequency of hiPSC-derived engineered heart tissues (hiPSC-EHTs) generated from control and PRDM16-overexpressing cardiomyocytes. Data are shown as mean ± SEM; ^∗∗^*p* < 0.01, n = 6–9 independent experiments. (J) Immunofluorescence staining of hiPSC-CMs for TNNI3 (red) with Hoechst nuclear counterstain (blue) under control and PRDM16-overexpressing conditions. Phase-contrast and merged images are shown. Scale bars, 200 μm. (K) Quantification of TNNI3 mean fluorescence intensity in control and PRDM16-overexpressing hiPSC-CMs. Data were determined by Welch’s *t* test and shown as mean ± SEM; ^∗^*p* < 0.05, *n* = 3 independent experiments. (L) Heatmap showing differential gene expression in PRDM16-overexpressing hiPSC-CMs relative to control, displayed as log fold change. Gene sets include cell-cycle regulators, cellular senescence markers, cytokinesis-associated genes, and genes involved in cardiomyocyte metabolic and structural maturation. (C), (F), and (H) show immunoblots derived from the same biological samples processed within a single Wes run; the β-actin loading control image is, therefore, shared between these three images.

To establish a causal relationship between PRDM16 expression and the cell cycle state of hiPSC-CMs and to determine whether PRDM16 facilitates the onset of developmental maturation, we ectopically overexpressed PRDM16 in hiPSC-CMs and monitored changes in proliferative dynamics benchmarked against known hallmarks of postnatal cardiomyocytes. To this end, we cloned the PRDM16 open reading frame (ORF) and inserted it as a cDNA into the lentiviral backbone pLenti6.3 (Figure 5A). As a control to assess transduction efficiency, we used pLenti6.3-GFP and optimized the viral titer to achieve robust PRDM16 overexpression (Figures S5A and S5B). Following successful lentiviral transduction, we confirmed a ∼6-fold increase in PRDM16 expression compared with control cells, both at mRNA (Figure 5B) and protein levels (Figure 5C).

Overexpression of PRDM16 resulted in a significant increase in cell size with no difference in the cell number (Figure 5D), indicative of cellular hypertrophy, a well-established hallmark of postnatal cardiomyocyte maturation in non-pathological settings (Johansson et al., 2020; Sakamoto and Kelly, 2024). Quantification of cell area across multiple microscopic fields confirmed that this hypertrophic phenotype was not only statistically significant but also showed a denser distribution of larger cells, with many doubling in size upon PRDM16 overexpression (Figure 5E).

Given that cardiomyocyte hypertrophy is typically inversely correlated with proliferation, we next assessed the expression of several key cell cycle regulators previously shown to be modulated by PRDM16 knockdown. PRDM16-overexpressing cells displayed a marked reduction in CDK1 and phospho-AKT protein levels (Figures 5F and S6C). In addition, we observed decreased phosphorylation of retinoblastoma protein (pRB1) and increased levels of p21, a cell cycle checkpoint inhibitor (Figures 5F and S6C). Immunostaining for MKI67 revealed a substantial reduction in proliferative activity in PRDM16-overexpressing cells (Figure S6B). Mitotic index analysis via pH3 staining confirmed a reduced mitotic rate (Figure S6A), reinforcing the role of PRDM16 in repressing cell cycle progression to the M-phase in developing cardiomyocytes.

To assess whether PRDM16 overexpression initiates a transition toward maturation, we examined the expression of the adult troponin isoform TNNI3. While *TNNI3* mRNA levels showed only a mild, non-significant increase (Figure 5G), protein analysis revealed a robust upregulation of TNNI3 in PRDM16-overexpressing cells (Figure 5H), suggesting post-transcriptional regulation and an RNA-protein mismatch. Mitochondrial OXPHOS proteins showed increased expression for complexes II, III, and V (Figure S5C), suggesting that energetic optimization resulting from PRDM16 overexpression might be more postnatal-like. Upon generating cardiac spheroids from hiPSC-CMs overexpressing PRDM16, we observed a significant reduction in beats per minute (BPM) (Figure 5I), a parameter commonly used as a proxy for cardiomyocyte maturation, reflecting decreased arrhythmogenicity and enhanced cellular maturation. ICC analysis further confirmed strong TNNI3 protein expression, supporting the idea that suppressed proliferation favors the onset of adult isoform expression in cardiomyocytes (Figures 5J, 5K, and S6C).

We next sequenced bulk-derived mRNA (RNA-seq) to assess global transcriptomic changes in pathways related to the proliferation-maturation axis studied. Overall, PRDM16 overexpression led to a significant reduction in proliferative genes involved in the cell cycle, extending beyond *CDK1* and *MKI67* to include other regulators such as *MCM2*, *AURKB*, *CCNB2*, and *CDC20* (Figure 5L). Loss of PRDM16 has recently been linked to senescent phenotypes and reduced tissue resilience (Cibi et al., 2020). To characterize the role of PRDM16-overexpressing cells in the context of senescence, we confirmed a marked downregulation of senescence mediators, including *CDKN2B*, *CXCL8*, and *MMP10* (Figure 5L). These findings suggest that PRDM16 contributes to cellular development and acquisition of new functions rather than driving biological aging or senescence. This observation may point to a possible antagonistic pleiotropic mechanism of PRDM16 in aging (Cibi et al., 2020).

In our model, PRDM16 expression defines a “Goldilocks zone,” where optimal expression decreases the proliferative capacity of hiPSC-CMs and, as a primary or secondary consequence, promotes metabolic, sarcomeric, and functional maturation of cardiac tissue. This is supported by increased levels of *MYH7* and *CD36* and decreased *HCN4* expression, among other markers (Figure 5L). Moreover, our model confirms that PRDM16 halts proliferation and cell division, particularly arresting cytokinesis (Figure 5L), as nuclear polyploidization with failed cytokinesis reflects maturation rather than immaturity (Gan et al., 2020).

Collectively, our results demonstrate that PRDM16 modulates the proliferative state of hiPSC-CMs by suppressing CDK1 and phospho-AKT, two critical growth and mitotic regulators. This repression establishes a transcriptional and functional environment conducive to maturation, as evidenced by enhanced expression of the adult cardiac troponin isoform TNNI3, consistent with the electrophysiological properties of a more mature myocardium.

## Discussion

Maturation of cardiomyocytes is essential for the functional development of adult myocardium; however, hiPSC-CMs differentiated *in vitro* typically retain fetal-like properties (Ahmed et al., 2020; Ernst et al., 2022; Iwoń et al., 2024; Koivumäki et al., 2018; Yuan et al., 2025). This immaturity limits their utility in disease modeling, drug discovery, and regenerative applications. Recently, PRDM16 has been recognized for its roles in developmental cardiology and metabolic regulation (Kodo et al., 2016). In this study, we investigated the function of PRDM16 in driving the transition from fetal-like to mature cardiomyocytes and examined the interplay between proliferative activity and maturation. Interestingly, Prdm16 protein has been reported to be downregulated postnatally in murine models and to contribute to cardiomyocyte proliferation (Wu et al., 2022). In contrast, our human data reveal a distinct trend. In hiPSC-derived cardiomyocytes, PRDM16 knockdown increases AURKB and pH3 levels (Figures 2H, 2J, 4F, and 4G), consistent with enhanced cell-autonomous proliferation under low PRDM16 conditions.

Our findings reveal that PRDM16 plays a pivotal role in the proliferative-to-maturation transition in cardiomyocytes. Notably, recent studies in model organisms have highlighted the spatiotemporal complexity of PRDM16 function. In zebrafish, *prdm16* loss-of-function mutants exhibit contractile dysfunction and dysregulated cardiomyocyte proliferation (Arndt et al., 2013), while in murine models, Prdm16 has been implicated in promoting cardiomyocyte proliferation during development (Wu et al., 2022). Together, these findings underscore that the role of PRDM16 in cardiomyocyte proliferation is highly context- and species-dependent. These apparent discrepancies between mouse and human systems may be explained by several, non-mutually exclusive scenarios. First, species-specific differences in developmental programs may underlie divergent PRDM16 functions. Second, RNA-protein discordance—frequently observed for transcriptional regulators—may obscure functional interpretation based solely on transcript levels. Third, PRDM16 may promote cardiomyocyte maturation through mechanisms such as nuclear polyploidization rather than by directly driving mitotic division. Fourth, PRDM16 function may be temporally regulated, initially supporting ventricular progenitor expansion and later promoting terminal maturation prior to its postnatal downregulation.

Collectively, these findings support a model in which PRDM16 acts as a bifunctional developmental switch in hiPSC-CMs. High PRDM16 levels promote the acquisition of adult-like structural and metabolic features while restraining proliferation, whereas reduced PRDM16 permits partial cell-cycle re-entry and reversion toward a more fetal-like phenotype. This model is consistent with PRDM16 being transiently engaged during ventricular progenitor expansion, followed by a role in terminal maturation, and ultimately downregulated after birth once cardiomyocyte number and architecture are established.

Loss- and gain-of-function experiments revealed that PRDM16 influences sarcomere organization, mitochondrial biogenesis, and cellular hypertrophy, while repressing proliferation-associated genes. Overexpression of PRDM16 was associated with upregulation of p21, suggesting partial cell-cycle arrest via a p21-dependent mechanism. Consistently, PRDM16 overexpression markedly reduced CDK1 expression while leaving CDK4 unchanged, indicating that PRDM16 preferentially regulates the G2/M transition rather than the G1/S checkpoint. Given CDK1’s critical role as a master checkpoint of mitosis, this reduction highlights PRDM16’s role in suppressing cell division. While cardiomyocyte maturation involves binucleation and polyploidization, reduced mitotic index (pH3^+^ cells) combined with decreased CDK1 provides stronger evidence that PRDM16 favors the shift toward maturation. This mechanism may involve direct regulation of HOPX, a transcription factor used as a proxy for maturation competence in murine models (Friedman et al., 2024), which we were able to validate in our human cardiogenesis platform. Thus, PRDM16 couples cell-cycle arrest with the initiation of structural and metabolic maturation programs.

Functionally, PRDM16-overexpressing hiPSC-CMs displayed hallmarks of maturation, including increased cell size, higher TNNI3 expression (Bedada et al., 2014), and reduced spontaneous beating frequency. These phenotypes align with molecular signatures enriched for mitochondrial genes and sarcomere components, further supporting the role of PRDM16 in coordinating both metabolic and structural maturation (Tian et al., 2025).

Previous strategies to induce hiPSC-CM maturation, such as metabolic substrate manipulation, electrical pacing, or hormone treatment, have achieved partial effects but often lacked integration between structural and metabolic programs (Feaster et al., 2015; Fetterman et al., 2024; Guo and Pu, 2020; Li et al., 2025; Feyen et al., 2020; Machiraju and Greenway, 2019). Our findings position PRDM16 as a regulator that synchronizes these domains, reminiscent of its role in brown adipose tissue differentiation (Harms et al., 2014), where it couples mitochondrial biogenesis to lineage-specific transcriptional programs.

These results have significant implications for generating more mature cardiac tissue models. PRDM16-overexpressing hiPSC-CMs may serve as improved platforms for pharmacological testing, particularly for drugs that target metabolism. Moreover, combining PRDM16 modulation with EHTs or three-dimensional organoid systems could yield more physiologically relevant models for preclinical applications. From a regenerative standpoint, PRDM16 expression or its modulation in immature cardiomyocytes may serve as a proxy in regeneration and may enhance engraftment efficiency and functional integration with host myocardium by preconditioning cells toward a more adult-like state.

Despite robust maturation phenotypes induced by PRDM16, some aspects of adult cardiomyocyte biology remain incompletely recapitulated. For example, although oxidative metabolism was enhanced, FAO did not reach adult-like levels. Furthermore, the direct genomic targets of PRDM16 in cardiomyocytes remain to be defined, and the epigenetic mechanisms underlying its long-term effects are still unclear. Future studies incorporating chromatin accessibility profiling and proteomics will be essential to map the PRDM16 interactome and establish its role in long-term cardiomyocyte maturation.

**Figure 6:**
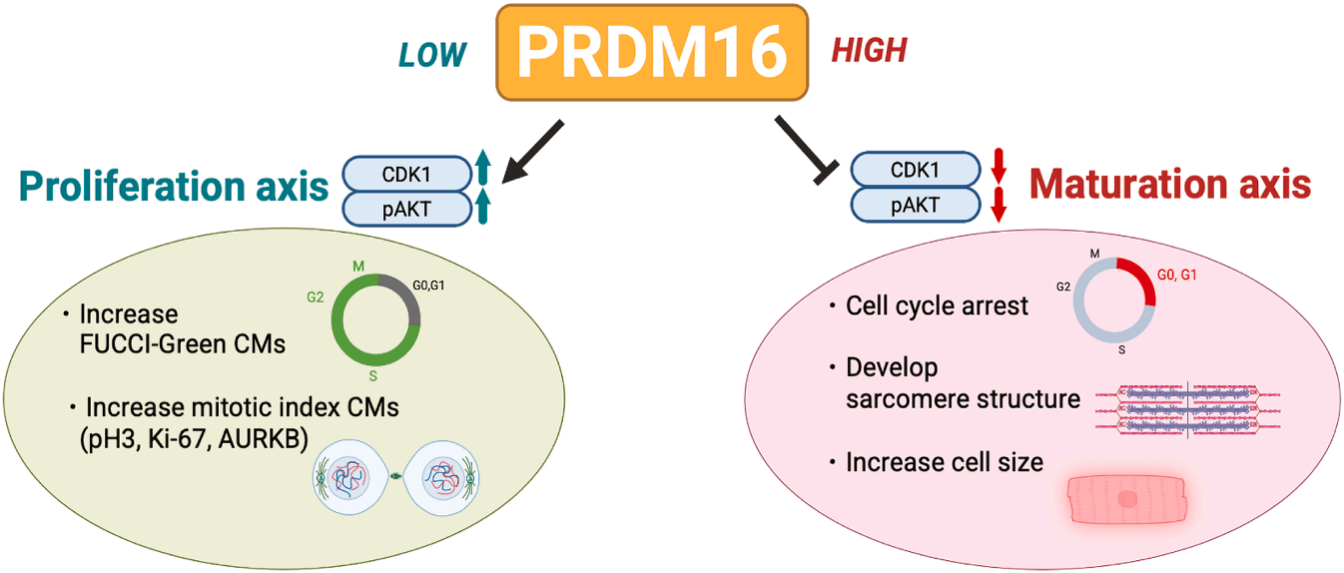
Model of PRDM16 as a regulator of the proliferation-maturation axis switch in hiPSC-CMs Schematic representation summarizing the dual role of PRDM16 in cardiomyocyte biology. Low PRDM16 expression promotes proliferation through upregulation of CDK1 and phospho-AKT and activation of metabolic programs associated with intrauterine-like states, resulting in increased mitotic index (pH3^+^, Ki-67^+^, and AURKB^+^) and sustained cell cycle activity. In contrast, high PRDM16 expression suppresses proliferation via CDK1 downregulation and phospho-AKT suppression, driving cardiomyocyte maturation characterized by cell-cycle arrest, organized sarcomere structure, and increased cell size.

In conclusion, PRDM16 contributes to modulating important aspects of cell cycle suppression and structural remodeling to facilitate cardiomyocyte maturation (Figure 6). Attenuation of PRDM16 elevated CDK1 and phosphorylated AKT, both critical mediators of cell growth and division, thereby promoting proliferation. In contrast, PRDM16 overexpression induced hypertrophic growth and sarcomeric remodeling, underscoring its dual role in structural adaptation and maturation. Altogether, the data indicate that PRDM16 influences the balance between proliferative and maturation programs during human cardiomyocyte development.

## Resource availability

### Lead contact

Further information and requests for resources and reagents should be directed to and will be fulfilled by the lead contact, Yoshinori Yoshida (yoshinor@cira.kyoto-u.ac.jp).

### Materials availability

All unique and stable reagents generated in this study are available from the lead contact upon completion of a materials transfer agreement.

### Data and code availability

All data presented in this study are available within the main text, in the supplemental information, and can be obtained from the corresponding authors upon request. The RNA-seq data have been deposited in the GEO database under the accession code GSE307782.

## Acknowledgments

We want to thank all members of the Yoshida laboratory for their constructive feedback on this study. This work was supported by a grant from the Leducq Foundation (18CVD05); JSPS KAKENHI grants (22K16137 to A.L.-C., 24K11267 to A.L.-C., and 21H02912 to Y.Y.); the Research Center Network for Realization of Regenerative Medicine, Japan Agency of Medical Research and Development (AMED) (JP19bm0104001 and JP20bm0804022 to Y.Y.); the Acceleration Program of R&D and implementation for Regenerative Medicine and Cell and Gene Therapy, AMED (JP23bm1423011, JP23bm1323001, and JP24bm1123054 to Y.Y.); the Research on Regulatory Science of Pharmaceuticals and Medical Devices, AMED (JP22mk0101241 and JP 24mk0121280 to Y.Y.); the Translational Research grant, AMED (JP22ym0126091 to Y.Y.); Program for Creating Deep Tech Startups with Global Reach, Japan Science and Technology Agency (JPMJSF2323 to Y.Y.); the IPS Cell Research Fund ( to A.L.-C. and Y.Y.); and JST SPRING (JPMJSP2110 to K.T.). We want to express our gratitude to Mikako Marx-Mori, Rumi Fujihara, Tomomi Gibson, and Hiroko Sata for their administrative support.

## Author contributions

A.L.-C. and Y.Y. conceived the study and designed experiments. K.T., Y.M., M.Nishikawa, and A.P.-E. performed experiments. M.Narita and Y.M. assisted in RNA-sequencing. A.L.-C., Y.Y., and K.T. analyzed and interpreted the data. Y.Y. and A.L.-C. provided funding and supervision. K.T., A.L.-C., and Y.Y. wrote the manuscript. All authors discussed the results.

## Declaration of interests

Y.Y. is a scientific advisor of Orizuru Therapeutics and receives grants from Takeda Pharmaceutical Company; MinebeaMitsumi; Sumitomo Bakelite; Altos Labs, Inc; and Toagosei Co., Ltd. outside the submitted work.

## Declaration of generative AI and AI-assisted technologies in the writing process

The authors acknowledge the use of ChatGPT solely for language editing and style refinement of the English text. After using this tool, the authors have reviewed and edited the content as needed and take full responsibility for the content of this publication.

## STAR★Methods

### Key resources table

**Table:** 

REAGENT or RESOURCE	SOURCE	IDENTIFIER
**Antibodies**		

Anti-Cardiac Troponin I antibody [4C2]	abcam	Cat#ab10231; RRID: AB_296967
Anti-TNNI1 antibody [EPR17120-11]	abcam	Cat#ab203515
Anti-Cardiac Troponin T antibody [1C11]	abcam	Cat#ab8295; RRID: AB_306445
Anti-Cardiac Troponin T antibody	abcam	Cat#ab45932; RRID: AB_956386
Anti-CDK1 antibody [EPR165]	abcam	Cat#ab133327; RRID: AB_11155333
Anti-p21 antibody [EPR362]	abcam	Cat#ab109520; RRID: AB_10860537
Anti-PRDM16 antibody	abcam	Cat#ab106410; RRID: AB_10866455
Anti-Aurora B antibody	abcam	Cat#ab2254; RRID: AB_302923
Total OXPHOS Human WB Antibody Cocktail	abcam	Cat#ab110411; RRID: AB_2756818
Anti-gamma H2A.X (phospho S139) antibody	abcam	Cat#ab11174; RRID: AB_297813
Anti-Ki67 antibody [SP6]	abcam	Cat#ab16667; RRID: AB_302459
CDK4 (D9G3E) Rabbit Monoclonal Antibody	CST	Cat#12790; RRID: AB_2631166
Phospho-Histone H3 (Ser10) Antibody	CST	Cat#9701; RRID: AB_331535
Phospho-Akt (Ser473) Antibody	CST	Cat#9271; RRID: AB_329825
Phospho-Rb (Ser807/811) (D20B12) Rabbit Monoclonal Antibody	CST	Cat#8516; RRID: AB_11178658
GAPDH (14C10) Rabbit Monoclonal Antibody	CST	Cat#2118; RRID: AB_561053
Anti-mouse IgG, HRP-linked Antibody	CST	Cat#7076; RRID: AB_330924
Anti-rabbit IgG, HRP-linked Antibody	CST	Cat#7074; RRID: AB_2099233
Anti-β-Actin (ACTB) Antibody	Sigma Aldrich	Cat#A5441; RRID: AB_476744
Goat anti-Mouse IgG (H + L) Superclonal™ Secondary Antibody, Alexa Fluor™ Plus 488	Thermo Fisher Scientific	Cat# A55058; RRID: AB_2921066
Goat anti-Rabbit IgG (H + L) Highly Cross-Adsorbed Secondary Antibody, Alexa Fluor™ 647	Thermo Fisher Scientific	Cat#A21245; RRID: AB_2535813
Donkey anti-Rabbit IgG (H + L) Highly Cross-Adsorbed Secondary Antibody, Alexa Fluor™ 594	Thermo Fisher Scientific	Cat#A21207; RRID: AB_141637
Alexa Fluor® 647 Mouse Anti-Cardiac Troponin T, Clone13-11	BD Pharmingen	Cat#565744; RRID: AB_2739341
Myosin Light Chain 2/MLC-2V Polyclonal antibody	Proteintech	Cat#55462-1-AP; RRID:AB_2881342

**Bacterial and virus strains**		

TFORF0675	Joung et al. (2023)	Addgene; Plasmid#141659
TFORF3549	Joung et al. (2023)	Addgene; Plasmid#145025

**Biological samples**		

Lenti-X 293T cells	Clontech	Cat#632180

**Chemicals, peptides, and recombinant proteins**		

Accumax	Innovative Cell Technologies	Cat#AM105
StemPro-34 medium	Invitrogen	Cat#10640019
Stempro-34 Supplement	Invitrogen	Cat#10641025
L-glutamine	Thermo Fisher Scientific	Cat#25030164
1-Thioglycerol	Sigma Aldrich	Cat#M6145
L-Ascorbic acid	Sigma Aldrich	Cat#A4544
Transferrin (Holo), from Human Blood	WAKO	Cat#204-18973
Y-27632	WAKO	Cat#036-24023
Matrigel Matrix Basement Membrane	Corning	Cat#354230
Recombinant Human BMP-4 Protein	R&D Systems	Cat#314-BP
Fibroblast Growth Factor (basic)	WAKO	Cat#060-04543
Recombinant Human/Mouse/Rat Activin A protein	R&D Systems	Cat#338-AC-010
Recombinant Human VEGF 165 Protein	R&D Systems	Cat#293-VE
Stemolecule Wnt Inhibitor IWP-3	REPROCELL USA Inc	Cat#04-0035
TrypLE Select Enzyme(1X)	Thermo Fisher Scientific	Cat#12563029
0.5mol/I-EDTA Solution (pH 8.0)	Nacalai tesque	Cat#06894-14
all-trans-Retinoic Acid	WAKO	Cat#188-01116
Silencer Select siRNA PRDM16	Thermo Fisher Scientific	Cat#4392420
Lipofectamine RNAiMAX	Thermo Fisher Scientific	Cat#13778150
Collagenase Type I	WAKO	Cat#035-17604
Torin1	Sigma Aldrich	Cat#475991
QIAzol Lysis Reagent	QIAGEN	Cat#79306
THUNDERBIRD Next SYBR qPCR Mix	TOYOBO	Cat#QPS-201
4% paraformaldehyde	WAKO	Cat#163-20145
Hoechst 33342	Thermo Fisher scientific	Cat#H3570
M-PER Mammalian Protein Extraction Reagent	Thermo Fisher scientific	Cat#0078501
MitoTracker™ Red CMXRos	Thermo Fisher scientific	Cat#M7512
Fibrinogen	Sigma Aldrich	Cat#F8630
Matrigel basement membrane	Corning	Cat#354234
Thrombin	Sigma Aldrich	Cat#T6884
Aprotinin	Sigma Aldrich	Cat#A1153
Insulin	WAKO	Cat#090-06481
Lenti-X concentrator	Clontech	Cat#631231
Polybrene	Merck	Cat#TR-1003-G

**Critical commercial assays**		

miRNeasy Micro Kit	QIAGEN	Cat#217084
qPCR RT Master Mix with gDNA Remover	TOYOBO	Cat#FSQ-301
Quick Start™ Bradford Protein Assay	Bio-Rad	Cat#5000207
Wes 12-230kDa Separation Modules	ProteinSimple	Cat#SM-W004A
Seahorse XF Cell Mito Stress Test Kit	Agilent Technology	Cat#103015-100
Lenti-X Packaging Single Shots	Clontech	Cat#631275
Lenti-X qRT-PCR Titration kit	Clontech	Cat#631235

**Deposited data**		

RNA-seq data	This study	GEO: GSE307782
Public RNA-seq data of FUCCI CMs	Kasamoto et al. (2023)	GEO: GSE176303

**Experimental models: Cell lines**		

Human iPSC	Kasamoto et al. (2023)	201B7-luc-FUCCI
Human iPSC	Center for iPS Cell Research and Application	1390D4

**Oligonucleotides**		

Primers for qPCR, See Table S1	This study	N/A

**Software and algorithms**		

GraphPad Prism software (v9 or later)	GraphPad	https://www.graphpad.com/scientific-software/prism/RRID: SCR_002798
The R Project for Statistical Computing (v4.2 or later)	R Core Team	https://www.r-project.org/RRID: SCR_001905
FlowJo v10.8.1	FlowJo	https://www.flowjo.com
Fiji (ImageJ v1.54m)	ImageJ	https://imagej.net RRID: SCR_002285

**Other**		

EHT silicone rack	DiNABIOS	N/A
Muscle Contraction Force Measurement Device	Sumitomo Bakelite Co., LTd.	BS-X9606

### Experimental model and study participant details

#### Human iPSC culture

The human induced pluripotent stem cell (iPSC) lines used in this study were 201B7 and 1390D4. The 201B7 iPSC line, engineered to express the Fluorescent Ubiquitination-based Cell Cycle Indicator (FUCCI) and luciferase (201B7-luc-FUCCI), was previously generated and characterized as described in a prior study (Kasamoto et al., 2023). The 1390D4 iPSC line was also utilized as an independent human iPSC model. Both cell lines were maintained under standard pluripotency culture conditions at 37°C in a humidified atmosphere containing 5% CO_2_. Cell line authentication and quality controls were monitored routinely.

#### Lenti-X 293T cell culture

Lenti-X 293T Cells (Clontech, 632180) were cultured in DMEM high glucose supplemented with 10% fetal bovine serum (FBS) and 0.5% penicillin-streptomycin The cells were maintained and incubated under standard culture conditions at 37°C in a humidified atmosphere with 5% CO2 until they reached 80%–90% confluency prior to lentiviral vector plasmid transfection.

### Method details

#### Cardiomyocyte differentiation from hiPSCs

For 201B7-luc-FUCCI, the cells were initially dissociated into single cells using Accumax (Innovative Cell Technologies, AM105) and subsequently seeded into poly (2-hydroxyethyl methacrylate)-coated 6 well plate at a density of 1×10^6^ cells per well. Cells were cultured in 1.5mL of StemPro-34 medium (Invitrogen, 10640019) with StemPro-34 supplement (Invitrogen, 10641025) enriched with L-glutamine (Thermo Fisher Scientific, 25030164), MTG (Sigma Aldrich, M6145), ascorbic acid (Sigma Aldrich, A4544), Transferrin (WAKO, 204–18973), the ROCK inhibitor Y-27632 (WAKO, 036–24023), Matrigel (Corning, 354230), and BMP4 (R&D Systems, 314-BP) to generate embryoid bodies (EBs). On the following day, an additional 1.5 mL medium containing bFGF (WAKO, 060–04543), activin A (R&D Systems, Inc, 338-AC-010), and BMP4, was added. On day 3, EBs were enzymatically dissociated again using Accumax and rinsed with IMDM (Thermo Fisher Scientific, 12440061) and resuspended in 2 mL the StemPro-34 medium supplemented with VEGF (R&D Systems, 293-VE), IWP-3 (REPROCELL USA Inc., 04–0035) and Matrigel. The medium was refreshed on day 7 and then replaced every 2–3 days thereafter. During the first 10 days, cells were cultured under hypoxic conditions (5% O_2_), after which they were shifted to a standard oxygen level.

The 1390D4 cell line was dissociated into a single-cell suspension using a 1:1 mixture of TrypLE Select (Thermo Fisher Scientific, 12563029) and 0.5 mM EDTA (Nacalai tesque, 06894-14), then cultured following a similar protocol to that used for 201B7-luc-FUCCI up to day 2. On day 3, cells were washed with IMDM and maintained in StemPro-34 medium containing VEGF and IWP-3 until day 7. Subsequently, the medium was switched to one supplemented with VEGF alone. After this stage, the culture conditions were aligned with those used for the 201B7-luc-FUCCI line. Retinoic acid (WAKO, 182–01116) was added on day 4 for atrial cardiomyocyte induction (Tsujisaka et al., 2022).

#### Knockdown experiment by siRNA

To suppress PRDM16 expression, we used a reverse transfection approach using siRNA PRDM16 (Thermo Fisher Scientific, 4392420). The transfection was carried out with Lipofectamine RNAiMAX (Thermo Fisher Scientific, 13778150). Cardiomyocytes were dissociated using Collagenase I (WAKO, 035–17604) and Accumax and subsequently plated onto 6-well plate at Day 16 in the presence of 5 pmol siRNA. The culture medium was replaced the following day. 72 h post-transfection, knockdown efficiency was assessed by SYBR-Green RT-qPCR. Upon confirmation of PRDM16 silencing, cells were treated with 200 nM Torin1 (Sigma Aldrich, 475991) for seven days.

#### Measurement of cell number

Day 25 monolayer cardiomyocytes were dissociated using Accumax for 10 min at 37°C. The cell suspension was diluted with FBS-supplemented IMDM and centrifuged at 800 rpm for 5 min. The resulting pellet was resuspended in culture medium, and viable cell numbers were determined using the Trypan Blue exclusion method with an automated cell counter (TC20, Bio-Rad). Fold changes were calculated after normalization to the control condition.

#### Bioinformatics and RNA-Sequencing

hiPSC-CM monolayers were collected at the indicated experimental time points by direct lysis in 700 μL of QIAzol Lysis Reagent (QIAGEN, 79306). Lysates were immediately stored at −80°C until RNA extraction. Total RNA was isolated using the miRNeasy Micro Kit (QIAGEN, 217084) according to the manufacturer’s instructions. RNA quality and concentration were assessed prior to library preparation and sequencing. For the in-house RNA-Seq data generated in this study, normalization was carried out with the NOISeq package. Both processed data and raw FASTQ files have been deposited in the Gene Expression Omnibus (GEO) under accession number GSE307782. Subsequent data analyses were performed in Rstudio.

Gene expression data were imported, explored, and pre-processed using NOISeq for differential expression analysis. To account for potential batch effects, batch correction was performed using the ComBat function (sva package) for the data presented in Figure 5L. Hierarchical clustering was conducted with the hclust function (stats package) and the agnes function (cluster package), applying the Manhattan (city-block) distance metric. K-means clustering was performed using the kmeans function. Distance and correlation matrices were computed and visualized using get_dist and fviz_dist (factoextra package), while cluster scatterplots were generated with fviz_cluster. Heatmaps were used to visualize differentially expressed genes (DEGs) with custom R scripts.

Functional enrichment and gene ontology (GO) clustering analyses were carried out using the DOSE and clusterProfiler packages for statistical assessment and visualization.

A retrospective analysis of FUCCI cardiomyocytes expression was performed using GSE176303 dataset.

#### Differential expression and bioinformatics analysis

Differential gene expression analysis was performed using the NOISeq package in R, which applies a non-parametric approach based on noise distribution modeling. Genes were considered differentially expressed using a probability threshold of q ≥ 0.8, as recommended by the NOISeq framework, together with a minimum absolute log2 fold change threshold where appropriate.

For visualization and downstream analyses: Principal component analysis (PCA) was performed using the prcomp function. Hierarchical clustering was conducted using Manhattan (city-block) distance and complete linkage. K-means clustering was applied using the kmeans function with optimized cluster number selection. Gene Ontology (GO) and pathway enrichment analyses were performed using clusterProfiler and DOSE packages. Multiple testing correction was performed using the Benjamini–Hochberg method, and terms with adjusted *p* value (FDR) < 0.05 were considered significant.

#### qRT-PCR

Cardiomyocytes were collected using 700 μL of QIAzol Lysis Reagent included in the miRneasy Micro Kit. Total RNA was extracted using the miRneasy Micro Kit according to the manufacturer’s instructions. The concentration of the extracted RNA was measured with a NanoDrop One, and the required amount was reverse transcribed into cDNA using the ReverTra Ace system (TOYOBO, FSQ301) following the manufacturer’s protocol. The cDNA was then used as a template for quantitative PCR (qPCR) with THUNDERBIRD Next SYBR qPCR Mix (TOYOBO, QPX-201) and specific primers. qPCR was performed on a QuantStudio 3 Real-Time PCR System (Applied Biosystems). Data were analyzed using the 2ˆ(−ΔΔCT) method and normalized to ACTB as the housekeeping gene. A complete list of primers is provided in Table S1.

#### Immunocytochemistry

On Day 16–17, cardiomyocytes were dissociated into single cells using Collagenase I and Accumax. Then, cells were plated onto 35mm glass bottom dish (Matsunami Glass, D11130H) precoated with fibronectin. Following seven days of culture with Torin1 treatment, cells were fixed with 4% paraformaldehyde (WAKO, 163–20145) for 15 min. Fixed cells were permeabilized and blocked for 30–45 min in PBS containing 1% BSA, 0.5% Triton X-100, 0.1M glycine.

For immunostaining, cells were incubated overnight at 4°C with the following primary antibodies in staining buffer (1% BSA, 0.5% Triton X-100 in PBS): Anti-Cardiac Troponin I (ab10231, Abcam, 1:200), anti-TNNI1 (ab203515, Abcam, 1:200), Anti-Cardiac Troponin T (ab45932, Abcam, 1:200), Phospho-Histone H3 (9701S, Cell Signaling Technology, 1:200), Gamma H2A.X (ab11174, Abcam, 1:200) AURKB (ab2254, Abcam, 1:200) and anti-human Ki-67 (ab16667, Abcam, 1:200). The next day, cells were washed three times with PBS and then incubated at room temperature for 2 h with the following secondary antibodies: Goat anti-mouse Alexa Fluor 488 (A55058, Thermo Fisher Scientific, 1:1000), Goat anti-rabbit Alexa Fluor 647 (A21245, Thermo Fisher Scientific, 1:1000), and Donkey anti-rabbit Alexa Fluor 594 (A21207, Thermo Fisher Scientific, 1:1000). After secondary incubation, cells were washed again three times with PBS.

For nuclear staining, Hoechst 33342 (H3570, Thermo Fisher Scientific, 1:10,000) was applied. Fluorescent images were acquired using an FV3000 confocal microscope (Olympus) and a BZ-X810 fluorescence microscope (KEYENCE).

#### Protein analysis

Cells were rinsed with ice-cold PBS and disrupted in M-PER Mammalian Protein Extraction Reagent (Thermo Fisher Scientific, 0078501) supplemented with protease and phosphatase inhibitors. The lysates were clarified by centrifugation at 12,000g for 15 min at 4°C, and only the supernatant was collected. Protein concentrations were determined using a Quick Start Bradford Protein Assay (Bio-Rad, 5000207) according to the manufacturer’s instructions and quantified with a EnVision 2104 plate reader (PerkinElmer). The measured protein samples were subsequently analyzed using a Wes 12-230kDa Separation Modules (ProteinSimple, SM-W004A). Data acquisition and quantification were performed with Compass for SW software v6.2.0 (ProteinSimple). A complete list of antibodies is provided in key resources table. Regarding Western blots, antibodies are included in the same table.

#### Mitotracker

For Flow cytometry, monolayer Day 26 CMs derived from 1390D4 were dissociated using Accumax and stained with 200nM MitoTracker Red CMXRos (Thermo Fisher Scientific, M7512) following by the manufacturer’s instructions. After staining, the cells were fixed with 4% paraformaldehyde and subsequently stained Cardiac Troponin T (565744, Becton Dickinson, 1000:1). Stained cells were analyzed by BD FACSAriaⅡand FlowJo software.

For immunocytochemistry, 15,000 Day16–17 CMs were seed onto a fibronectin-coated 35mm glass bottom dish. After one week of treatment, cells were stained with 200nM MitoTracker Red CMXRos following the manufacturer’s instructions. Fluorescent images were acquired using FV3000 confocal microscope (Olympus).

#### Seahorse mitostress test

The mitochondrial stress test (Agilent Technologies, 103015-100) was performed using a Seahorse XF Pro extracellular flux analyzer (Agilent Technologies).

One day prior to the assay, 20,000 Day 26 CMs were seeded onto a fibronectin-coated Seahorse XF cell culture microplate. CMs were prepared according to the manufacturer’s instructions. The concentrations of compounds were optimized for these cells as follows; 1.5μM Oligomycin, 2.0μM FCCP and 0.5μM Rotenone + Antimycin A. Data was analyzed by Wave Pro software (Agilent Technologies).

#### Generation of EHT

On day16 of beating EBs (with 70–80% cTNT-positive cells) differentiated from the on-feeder human iPS cell line 201B7-luc-FUCCI were dissociated using Collagenase I overnight. The following day, a single-cell suspension was obtained by a second enzymatic digestion with Accumax at 37°C for 15 min. After dissociation, the cells were resuspended in DMEM supplemented with 1% penicillin/streptomycin and 10% Fetal Bovine Serum (FBS). EHTs were generated with EHT silicone rack (DiNABIOS) and EHT device (Sumitomo Bakelite Co., Ltd.) in a 24 well plate format. A total of 1 × 10^6^ cells were mixed with an EHT Master mix (NCM medium, fibrinogen (Sigma Aldrich, F8630), Matrigel basement membrane (Corning, 354234), 2x DMEM and Y-27632). For each EHT, 97 μL of the master mix was combined with 3 μL of thrombin (Sigma Aldrich, T6884) and the mixture was pipetted into the EHT device placed in the silicone rack and incubated at 37°C. After one hour incubation, the silicone rack with attached EHT were transferred to 24 well plate containing EHT medium (DMEM, heat-inactivated bovine serum, penicillin/streptomycin, aprotinin (Sigma Aldrich, A1153), and insulin (WAKO, 090–06481)). The culture medium was changed every other day starting two days after EHT generation.

#### Measurement of contractile force

When we measure the contractile force, EHT is paced using C-Pace EM (1.0Hz, 5ms, 5V). Taking the movie of contraction for 15 s is by BZ-X800 (KEYENCE). Contractile force was calculated using ImageJ and used the following formula.

$$\text{Contractile}\;\text{force}\;\left(N\right)=3\times 3.14\times {\text{ER}}^{4}\times \left(\text{distance}\left(\text{mm}\right)\right)/64\times L^{3}$$

Contractileforce(N/mm2)=P3.14×(Tissuewidth2)2

E = 1.7 (Mpa; young module), R = 1.0 (mm; diameter of poles), L = 12(mm; length of poles).

To get the length, we picked up 3 contractions and pointed the maximum diastole, the maximum systole and the width. Each parameter is introduced in the formula and got the contractile force.

#### Overexpression of PRDM16

As PRDM16 plasmid, lentiviral vector which is named TFORF0675 (#141659) was applied to overexpression and TFORF3549 (#145025) was used for the control. (Joung et al., 2023).

Using 80–90% confluent lenti-X 293T cells (Clontech), 7 μg lenti virus vector plasmid and Lenti-X Packaging Single Shots (Clontech, 631275) were mixed and added to them. Incubated cells overnight and added fresh medium and incubate them for 48 h. After incubation, collect the virus liquid and added Lenti-X concentrator (Clontech, 631231) to get high titration. To measure the titer, Lenti-X qRT-PCR Titration kit (Clontech, 631235) was used and got copy number.

For overexpression PRDM16, CMs derived from iPSC were seeded on 12 well plate and performed transduction with purified viruses. When transduction is performed, 4 μg/ml Polybrene (Merck, TR-1003-G) was added and used 4,000 copies/cell in our study.

#### Data visualization and figure preparation

Schematic illustrations were created using BioRender.com and finalized using Adobe Illustrator.

### Quantification and statistical analysis

#### Statistical analysis

All statistical analyses were performed using GraphPad Prism (v9 or later) and R (v4.2 or later). Unless otherwise stated, data are presented as mean ± standard error of the mean (SEM) from at least three independent differentiation experiments.

For comparisons between two groups, statistical significance was assessed using an unpaired two-tailed Welch’s *t* test. For comparisons involving more than two groups, one-way ANOVA followed by Tukey’s post hoc test was applied. When two independent variables were analyzed (e.g., treatment and genetic perturbation), two-way ANOVA followed by appropriate post hoc multiple comparison tests was used.

Normality of data distribution was assessed using the Shapiro–Wilk test, and variance homogeneity was evaluated using Levene’s test. In cases where data did not meet parametric assumptions, non-parametric tests (Mann–Whitney U test for two groups or Kruskal–Wallis test with Dunn’s correction for multiple comparisons) were applied.

For all analyses, a *p* value <0.05 was considered statistically significant. Exact *p* values and statistical tests used are indicated in the corresponding figure legends.

## Supplemental information

Document S1. Figures S1–S6 and Table S1

Document S2. Article plus supplemental information

## References

[bib1] Aguilo F., Avagyan S., Labar A., Sevilla A., Lee D.-F., Kumar P., Lemischka I.R., Zhou B.Y., Snoeck H.-W. (2011). Prdm16 is a physiologic regulator of hematopoietic stem cells. Blood.

[bib2] Ahmed R.E., Anzai T., Chanthra N., Uosaki H. (2020). A Brief Review of Current Maturation Methods for Human Induced Pluripotent Stem Cells-Derived Cardiomyocytes. Front. Cell Dev. Biol..

[bib3] Arndt A.K., Schafer S., Drenckhahn J.D., Sabeh M.K., Plovie E.R., Caliebe A., Klopocki E., Musso G., Werdich A.A., Kalwa H. (2013). Fine Mapping of the 1p36 Deletion Syndrome Identifies Mutation of PRDM16 as a Cause of Cardiomyopathy. Am. J. Hum. Genet..

[bib4] Bedada F.B., Chan S.S.-K., Metzger S.K., Zhang L., Zhang J., Garry D.J., Kamp T.J., Kyba M., Metzger J.M. (2014). Acquisition of a quantitative, stoichiometrically conserved ratiometric marker of maturation status in stem cell-derived cardiac myocytes. Stem Cell Rep..

[bib5] Biferali B., Bianconi V., Perez D.F., Kronawitter S.P., Marullo F., Maggio R., Santini T., Polverino F., Biagioni S., Summa V. (2021). Prdm16-mediated H3K9 methylation controls fibro-adipogenic progenitors identity during skeletal muscle repair. Sci. Adv..

[bib6] Cerneckis J., Cai H., Shi Y. (2024). Induced pluripotent stem cells (iPSCs): molecular mechanisms of induction and applications. Signal Transduct. Target. Ther..

[bib7] Cibi D.M., Bi-Lin K.W., Shekeran S.G., Sandireddy R., Tee N., Singh A., Wu Y., Srinivasan D.K., Kovalik J.-P., Ghosh S. (2020). Prdm16 Deficiency Leads to Age-Dependent Cardiac Hypertrophy, Adverse Remodeling, Mitochondrial Dysfunction, and Heart Failure. Cell Rep..

[bib8] Clancy C.E., Santana L.F. (2024). Advances in induced pluripotent stem cell-derived cardiac myocytes: technological breakthroughs, key discoveries and new applications. J. Physiol..

[bib9] Ernst P., Bidwell P.A., Dora M., Thomas D.D., Kamdar F. (2022). Cardiac calcium regulation in human induced pluripotent stem cell cardiomyocytes: Implications for disease modeling and maturation. Front. Cell Dev. Biol..

[bib10] Feaster T.K., Cadar A.G., Wang L., Williams C.H., Chun Y.W., Hempel J.E., Bloodworth N., Merryman W.D., Lim C.C., Wu J.C. (2015). Matrigel Mattress: A Method for the Generation of Single Contracting Human-Induced Pluripotent Stem Cell-Derived Cardiomyocytes. Circ. Res..

[bib11] Fetterman K.A., Blancard M., Lyra-Leite D.M., Vanoye C.G., Fonoudi H., Jouni M., DeKeyser J.-M.L., Lenny B., Sapkota Y., George A.L., Burridge P.W. (2024). Independent compartmentalization of functional, metabolic, and transcriptional maturation of hiPSC-derived cardiomyocytes. Cell Rep..

[bib29] Feyen D.A.M., McKeithan W.L., Bruyneel A.A.N., Spiering S., Hörmann L., Ulmer B., Zhang H., Briganti F., Schweizer M., Hegyi B. (2020). Metabolic Maturation Media Improve Physiological Function of Human iPSC-Derived Cardiomyocytes. Cell Rep..

[bib12] Friedman C.E., Cheetham S.W., Negi S., Mills R.J., Ogawa M., Redd M.A., Chiu H.S., Shen S., Sun Y., Mizikovsky D. (2024). HOPX-associated molecular programs control cardiomyocyte cell states underpinning cardiac structure and function. Dev. Cell.

[bib13] Gan P., Patterson M., Sucov H.M. (2020). Cardiomyocyte Polyploidy and Implications for Heart Regeneration. Annu. Rev. Physiol..

[bib14] Garbern J.C., Helman A., Sereda R., Sarikhani M., Ahmed A., Escalante G.O., Ogurlu R., Kim S.L., Zimmerman J.F., Cho A. (2020). Inhibition of mTOR Signaling Enhances Maturation of Cardiomyocytes Derived From Human-Induced Pluripotent Stem Cells via p53-Induced Quiescence. Circulation.

[bib15] Goldfracht I., Efraim Y., Shinnawi R., Kovalev E., Huber I., Gepstein A., Arbel G., Shaheen N., Tiburcy M., Zimmermann W.H. (2019). Engineered heart tissue models from hiPSC-derived cardiomyocytes and cardiac ECM for disease modeling and drug testing applications. Acta Biomater..

[bib16] Gudmundsson K.O., Nguyen N., Oakley K., Han Y., Gudmundsdottir B., Liu P., Tessarollo L., Jenkins N.A., Copeland N.G., Du Y. (2020). Prdm16 is a critical regulator of adult long-term hematopoietic stem cell quiescence. Proc. Natl. Acad. Sci. USA.

[bib17] Guo Y., Pu W.T. (2020). Cardiomyocyte Maturation: New Phase in Development. Circ. Res..

[bib18] Harms M.J., Ishibashi J., Wang W., Lim H.-W., Goyama S., Sato T., Kurokawa M., Won K.-J., Seale P. (2014). Prdm16 is required for the maintenance of brown adipocyte identity and function in adult mice. Cell Metab..

[bib19] He L., Wen J., Dai Q. (2025). PRDM16 functions as a co-repressor in the BMP pathway to suppress neural stem cell proliferation. eLife.

[bib20] Iismaa S.E., Kaidonis X., Nicks A.M., Bogush N., Kikuchi K., Naqvi N., Harvey R.P., Husain A., Graham R.M. (2018). Comparative regenerative mechanisms across different mammalian tissues. npj Regen. Med..

[bib21] Iwoń Z., Krogulec E., Tarnowska I., Łopianiak I., Wojasiński M., Dobrzyń A., Jastrzębska E. (2024). Maturation of human cardiomyocytes derived from induced pluripotent stem cells (iPSC-CMs) on polycaprolactone and polyurethane nanofibrous mats. Sci. Rep..

[bib22] Johansson M., Ulfenborg B., Andersson C.X., Heydarkhan-Hagvall S., Jeppsson A., Sartipy P., Synnergren J. (2020). Cardiac hypertrophy in a dish: a human stem cell based model. Biol. Open.

[bib23] Joung J., Ma S., Tay T., Geiger-Schuller K.R., Kirchgatterer P.C., Verdine V.K., Guo B., Arias-Garcia M.A., Allen W.E., Singh A. (2023). A transcription factor atlas of directed differentiation. Cell.

[bib24] Karakikes I., Ameen M., Termglinchan V., Wu J.C. (2015). Human Induced Pluripotent Stem Cell-Derived Cardiomyocytes: Insights into Molecular, Cellular, and Functional Phenotypes. Circ. Res..

[bib25] Kasamoto M., Funakoshi S., Hatani T., Okubo C., Nishi Y., Tsujisaka Y., Nishikawa M., Narita M., Ohta A., Kimura T., Yoshida Y. (2023). Am80, a retinoic acid receptor agonist, activates the cardiomyocyte cell cycle and enhances engraftment in the heart. Stem Cell Rep..

[bib26] Kodo K., Ong S.-G., Jahanbani F., Termglinchan V., Hirono K., InanlooRahatloo K., Ebert A.D., Shukla P., Abilez O.J., Churko J.M. (2016). iPSC-derived cardiomyocytes reveal abnormal TGFβ signaling in left ventricular non-compaction cardiomyopathy. Nat. Cell Biol..

[bib27] Koivumäki J.T., Naumenko N., Tuomainen T., Takalo J., Oksanen M., Puttonen K.A., Lehtonen Š., Kuusisto J., Laakso M., Koistinaho J., Tavi P. (2018). Structural Immaturity of Human iPSC-Derived Cardiomyocytes: In Silico Investigation of Effects on Function and Disease Modeling. Front. Physiol..

[bib28] Li W., Luo X., Strano A., Arun S., Gamm O., Poetsch M.S., Hasse M., Steiner R.-P., Fischer K., Pöche J. (2025). Comprehensive promotion of iPSC-CM maturation by integrating metabolic medium with nanopatterning and electrostimulation. Nat. Commun..

[bib30] Machiraju P., Greenway S.C. (2019). Current methods for the maturation of induced pluripotent stem cell-derived cardiomyocytes. World J. Stem Cells.

[bib31] Paik D.T., Chandy M., Wu J.C. (2020). Patient and Disease–Specific Induced Pluripotent Stem Cells for Discovery of Personalized Cardiovascular Drugs and Therapeutics. Pharmacol. Rev..

[bib32] Sakamoto T., Kelly D.P. (2024). Cardiac maturation. J. Mol. Cell. Cardiol..

[bib33] Satoh M., Ohkubo T., Miura K., Harada A., Tsutsui A., Hozawa A., Shimizu Y., Ishikawa S., Kokubo Y., Okamura T. (2025). Long-term risk of cardiovascular mortality according to age group and blood pressure categories of the latest guideline. Hypertens. Res..

[bib34] Sun B., Rouzbehani O.M.T., Kramer R.J., Ghosh R., Perelli R.M., Atkins S., Fatahian A.N., Davis K., Szulik M.W., Goodman M.A. (2023). Nonsense Variant PRDM16-Q187X Causes Impaired Myocardial Development and TGF-β Signaling Resulting in Noncompaction Cardiomyopathy in Humans and Mice. Circ. Heart Fail..

[bib35] Tani H., Tohyama S. (2022). Human Engineered Heart Tissue Models for Disease Modeling and Drug Discovery. Front. Cell Dev. Biol..

[bib36] Thompson M., Sakabe M., Verba M., Hao J., Meadows S.M., Lu Q.R., Xin M. (2023). PRDM16 regulates arterial development and vascular integrity. Front. Physiol..

[bib37] Tian Y., Tsujisaka Y., Li V.Y., Tani K., Lucena-Cacace A., Yoshida Y. (2022). Immunosuppressants Tacrolimus and Sirolimus revert the cardiac antifibrotic properties of p38-MAPK inhibition in 3D-multicellular human iPSC-heart organoids. Front. Cell Dev. Biol..

[bib38] Tian Y., Lucena-Cacace A., Tani K., Elvandari A.P., Allendes Osorio R.S., Narita M., Matsumura Y., Paixao I.C., Miyoshi Y., Inagaki A. (2025). Generation of mature epicardium derived from human-induced pluripotent stem cells via inhibition of mTOR signaling. Nat. Commun..

[bib39] Tsujisaka Y., Hatani T., Okubo C., Ito R., Kimura A., Narita M., Chonabayashi K., Funakoshi S., Lucena-Cacace A., Toyoda T. (2022). Purification of human iPSC-derived cells at large scale using microRNA switch and magnetic-activated cell sorting. Stem Cell Rep..

[bib40] Van Wauwe J., Kemps H., Vrancaert P., Mahy A., Schellingen R., Grootaert M.O.J., Beerens M., Luttun A. (2025). PRDM16, a new kid on the block in cardiovascular health and disease. Cardiovasc. Res..

[bib41] Wang Z., Zhao X., Zhao G., Guo Y., Lu H., Mu W., Zhong J., Garcia-Barrio M., Zhang J., Chen Y.E., Chang L. (2023). PRDM16 deficiency in vascular smooth muscle cells aggravates abdominal aortic aneurysm. JCI Insight.

[bib42] Wu T., Liang Z., Zhang Z., Liu C., Zhang L., Gu Y., Peterson K.L., Evans S.M., Fu X.-D., Chen J. (2022). PRDM16 Is a Compact Myocardium-Enriched Transcription Factor Required to Maintain Compact Myocardial Cardiomyocyte Identity in Left Ventricle. Circulation.

[bib43] Yuan Q., Verbueken D., Dinani R., Kim R., Schoger E., Morsink C.D., Simkooei S.A., Kemna L.J.M., Hjortnaes J., Kuster D.W.D. (2025). Glycogen synthase kinase-3 inhibition and insulin enhance proliferation and inhibit maturation of human iPSC-derived cardiomyocytes via TCF and FOXO signaling. Stem Cell Rep..

[bib44] Zhao S., Nie T., Li L., Long Q., Gu P., Zhang Y., Sun W., Lin Z., Liu Q., Qi Y. (2023). Androgen Receptor is a Negative Regulator of PRDM16 in Beige Adipocyte. Adv. Sci..

