# Supplementary material for: PRDM16 modulates aspects of cell cycle dynamics and maturation in human iPSC-derived cardiomyocytes
Source: Stem Cell Reports. 2026 Jul 9;21(8):103005. doi: 10.1016/j.stemcr.2026.103005 (PMC13476894; doi:10.1016/j.stemcr.2026.103005)
Supplement: Supplementary file 1 [file mmc1.pdf]

Stem Cell Reports, Volume *21*

Supplemental Information

PRDM16 modulates aspects of cell cycle dynamics and maturation in human iPSC-derived cardiomyocytes

Kanae Tani, Yasuko Matsumura, Misato Nishikawa, Megumi Narita, Amanda Putri Elvandari, Antonio Lucena-Cacace, and Yoshinori Yoshida

SUPPLEMENTAL INFORMATION

Figure S1

Figure S1

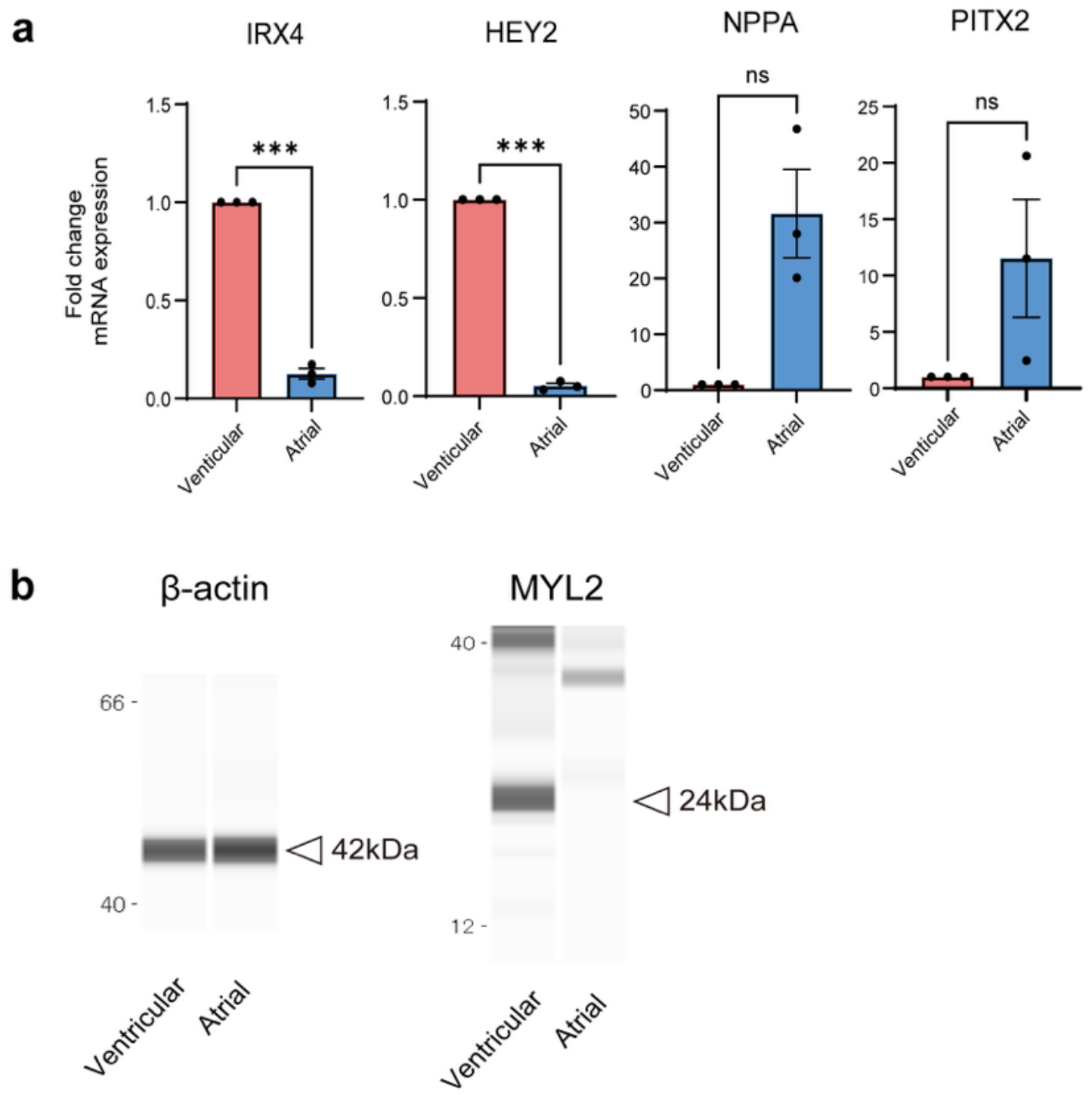

Figure S2

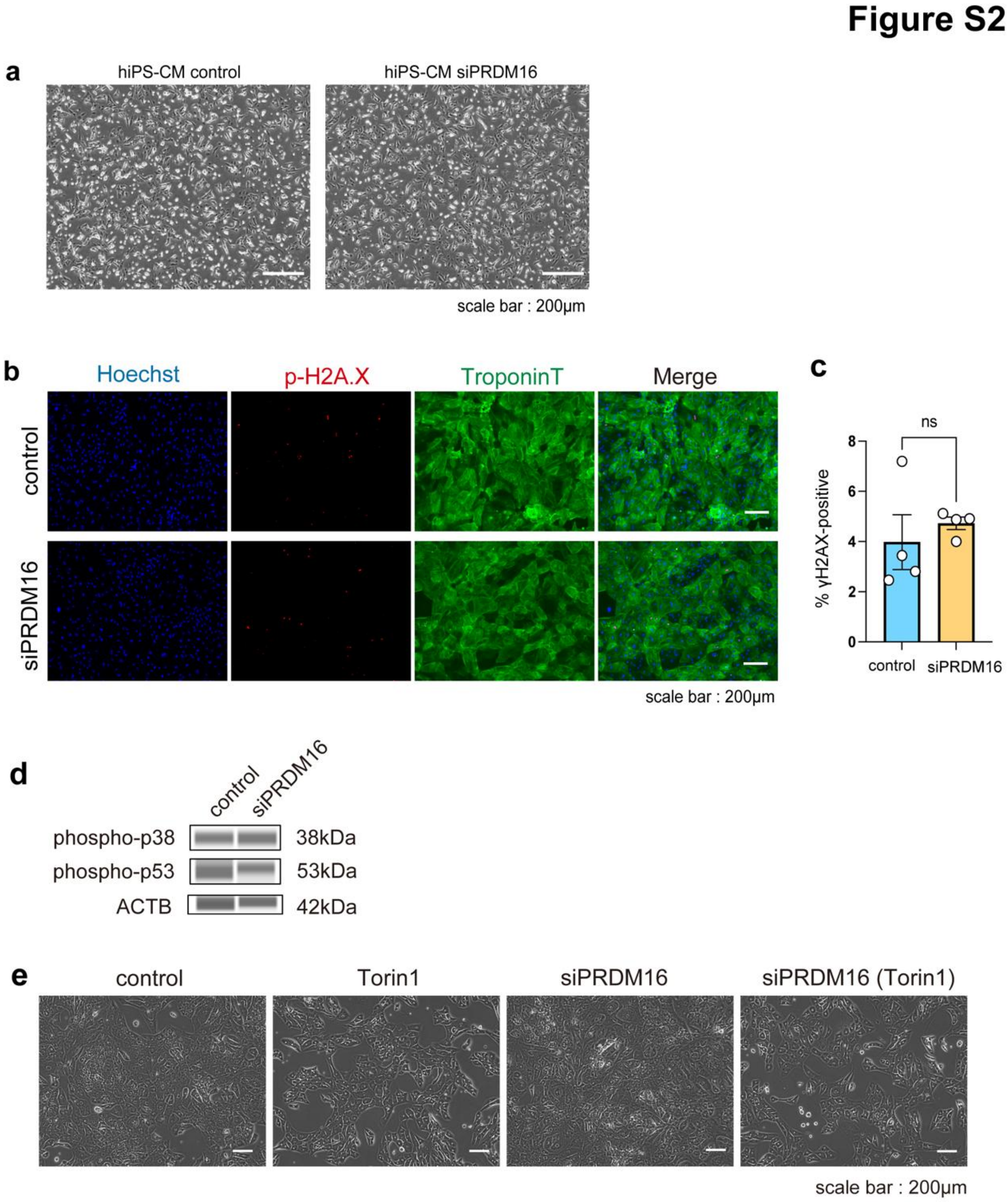

Figure S2
a
hiPS-CM control
hiPS-CM siPRDM16
scale bar : 200μm
b
Hoechst
p-H2A.X
TroponinT
Merge
control
siPRDM16
scale bar : 200μm
c
ns
% γH2AX-positive
8
6
4
2
0
control
siPRDM16
d
control
siPRDM16
phospho-p38
38kDa
phospho-p53
53kDa
ACTB
42kDa
e
control
Torin1
siPRDM16
siPRDM16 (Torin1)
scale bar : 200μm

Figure S3

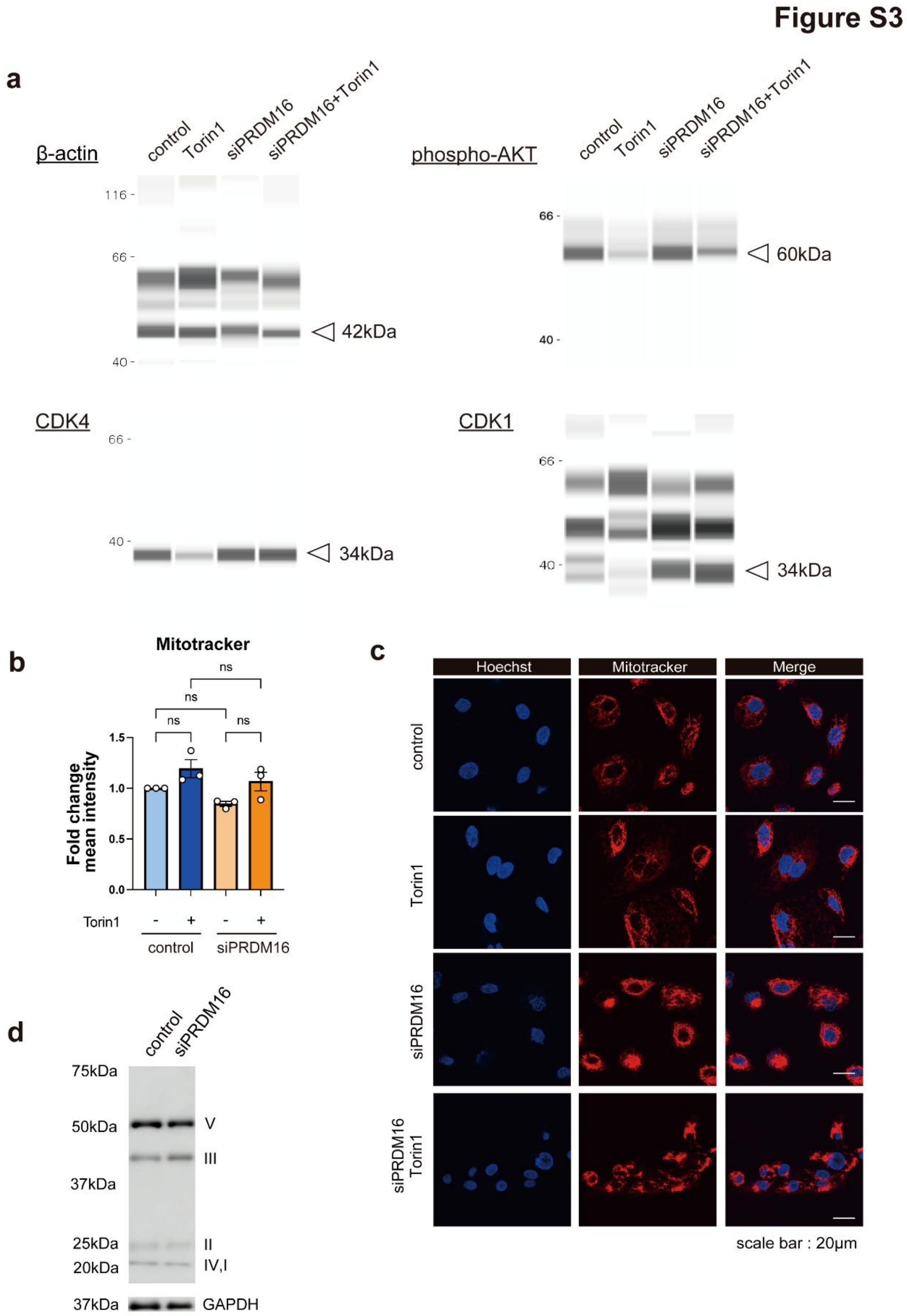
Figure S3
a
β-actin
control
Torin1
siPRDM16
siPRDM16+Torin1
116
66
40
42kDa
phospho-AKT
66
40
60kDa
CDK4
66
40
34kDa
CDK1
66
40
34kDa
b
Mitotracker
ns
Fold change
mean intensity
1.5
1.0
0.5
0.0
Torin1
-
+
control
siPRDM16
c
Hoechst
Mitotracker
Merge
control
Torin1
siPRDM16
siPRDM16
Torin1
scale bar : 20μm
d
control
siPRDM16
75kDa
50kDa
37kDa
25kDa
20kDa
V
III
II
IV,I
37kDa
GAPDH

Figure S4

Figure S4

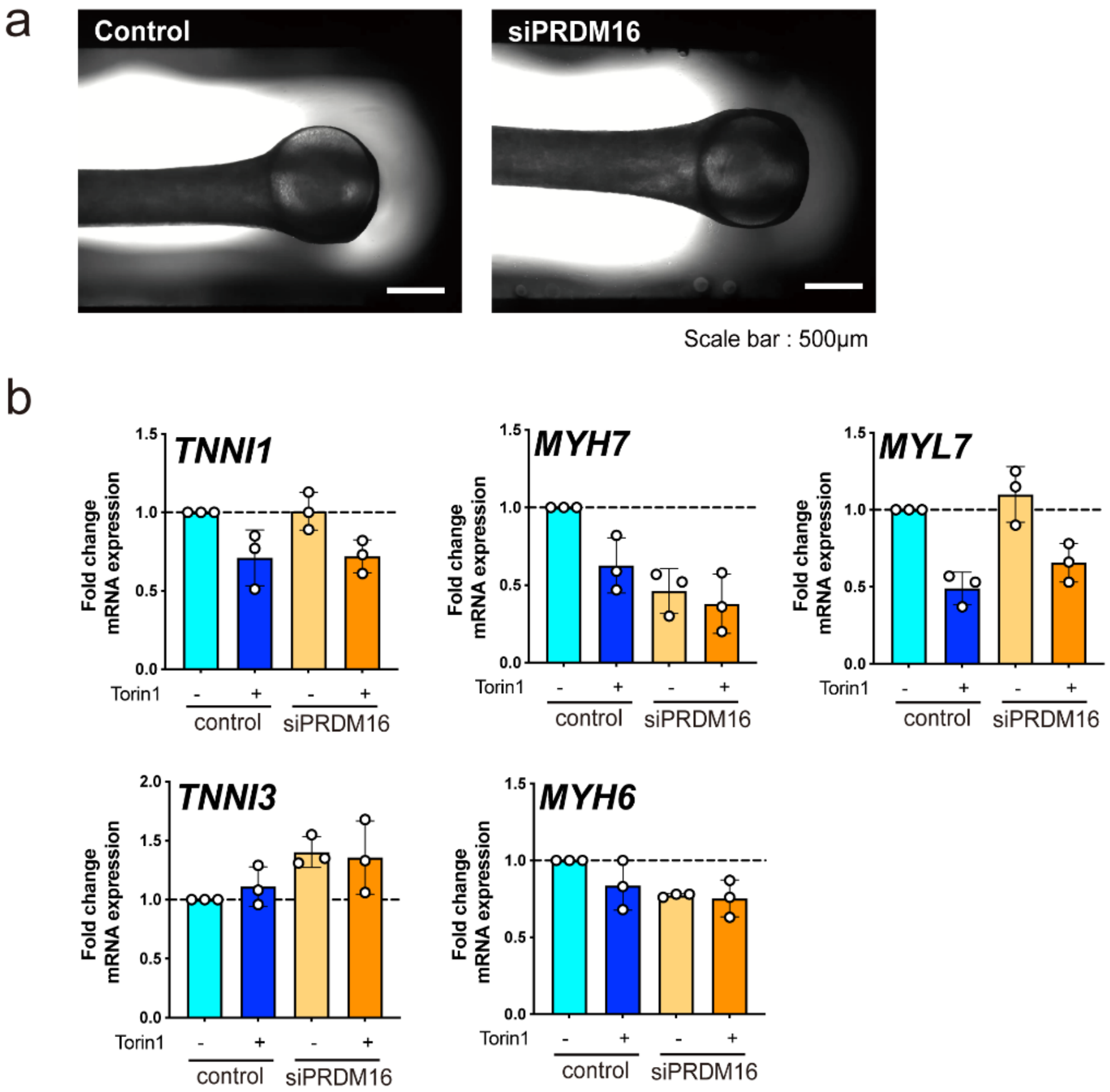

Figure S5

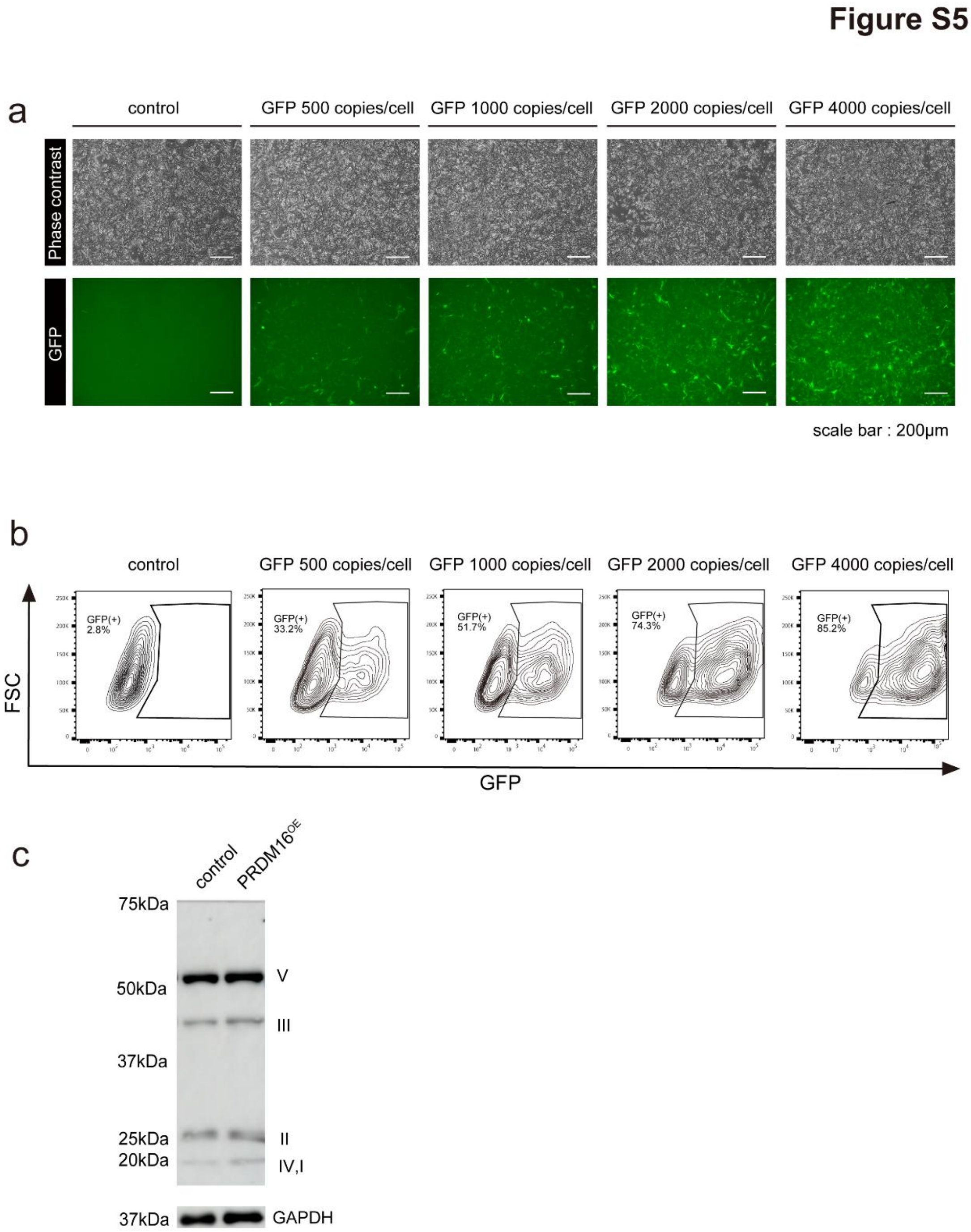
Figure S5
a
control
GFP 500 copies/cell
GFP 1000 copies/cell
GFP 2000 copies/cell
GFP 4000 copies/cell
Phase contrast
GFP
scale bar : 200μm
b
control
GFP 500 copies/cell
GFP 1000 copies/cell
GFP 2000 copies/cell
GFP 4000 copies/cell
GFP(+) 2.8%
GFP(+) 33.2%
GFP(+) 51.7%
GFP(+) 74.3%
GFP(+) 85.2%
FSC
GFP
c
control
PRDM16OE
75kDa
50kDa
37kDa
25kDa
20kDa
37kDa
V
III
II
IV,I
GAPDH

Figure S6

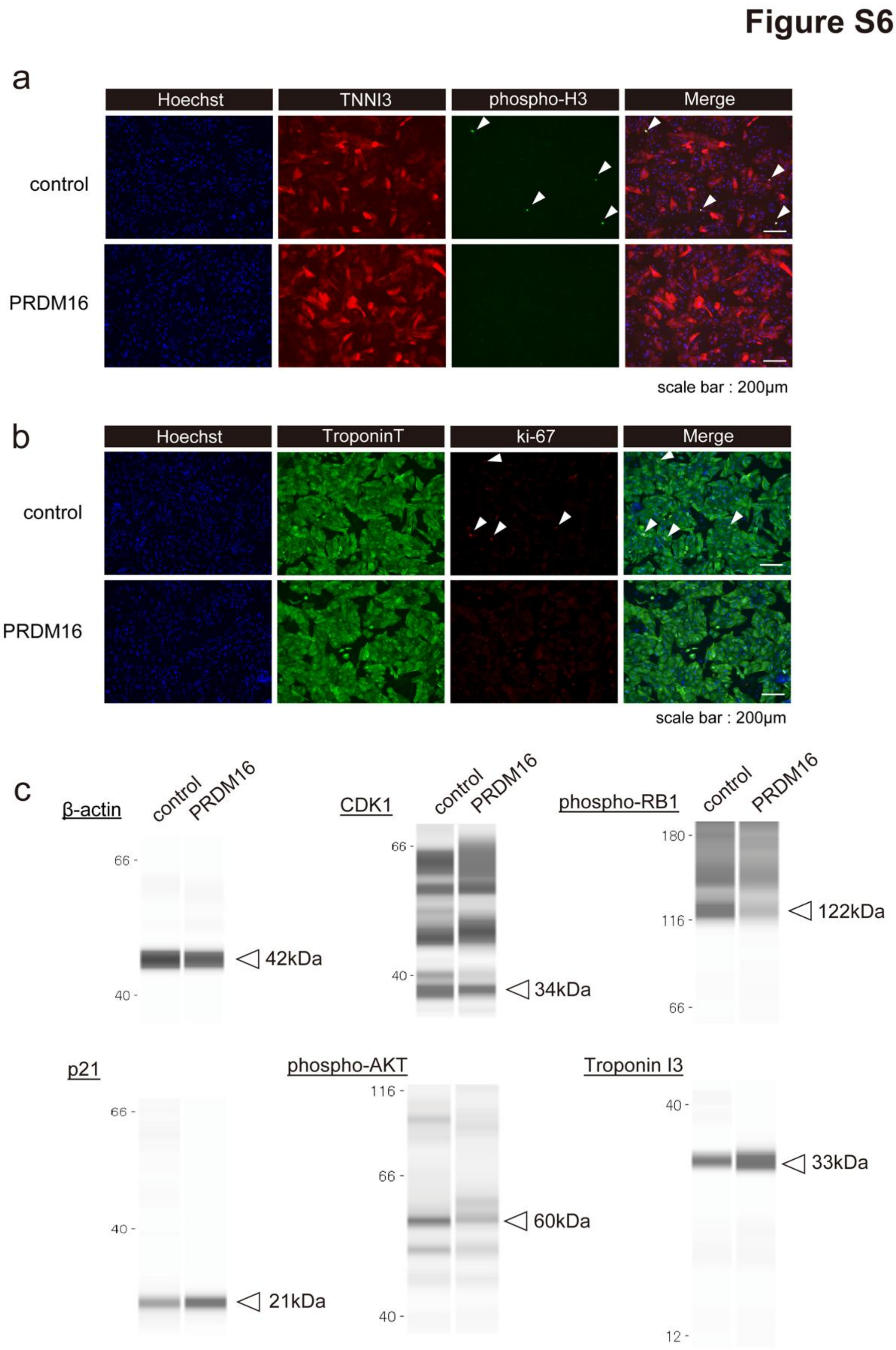
Figure S6
a
Hoechst
TNNI3
phospho-H3
Merge
control
PRDM16
scale bar : 200μm
b
Hoechst
TroponinT
ki-67
Merge
control
PRDM16
scale bar : 200μm
c
β-actin
control
PRDM16
66
40
42kDa
CDK1
control
PRDM16
66
40
34kDa
phospho-RB1
control
PRDM16
180
116
66
122kDa
p21
66
40
21kDa
phospho-AKT
116
66
40
60kDa
Troponin I3
40
12
33kDa

SUPPLEMENTARY FIGURE LEGEND

Figure S1. The identification of ventricular cardiomyocytes. (related to Figure 1)

(a) Quantitative RT–PCR analysis of ventricular (IRX4, HEY2) and atrial (NPPA, PITX2) marker expression in ventricular versus atrial hiPSC-derived cardiomyocytes. Data are shown as mean ± SEM; ns, not significant; ***P < 0.001, n=3 independent cultures per condition.

(b) Immunoblot analysis of ventricular marker (MYL2), using β-actin as a loading control.

Figure S2. The effect of transfected siPRDM16 in hiPS-CMs. (related to Figure 2)

(a) Representative phase-contrast images of hiPSC-CMs under control and siPRDM16 conditions. Scale bar, 200 μm.

(b) Immunofluorescence staining of hiPSC-CMs for phospho-H2AX (γH2AX) (red) and cardiac troponin T (TNNT2) (green), with Hoechst nuclear counterstain (blue), under control and siPRDM16 conditions. Scale bar, 200 μm.

(c) Quantification of $\gamma H2AX^{+}$ cardiomyocytes shown in (b). Data are presented as mean ± SEM; ns, not significant, n=4 independent experiments.

(d) Immunoblot analysis of stress-response signaling pathways, showing phosphorylated p38 MAPK and phosphorylated p53 in control and siPRDM16 hiPSC-CMs. ACTB serves as a loading control.

(e) Representative phase-contrast images of hiPSC-CM monolayers under control, Torin1, siPRDM16, and siPRDM16 + Torin1 conditions. Scale bar, 200 μm.

Figure S3. Mitochondrial abundance, distribution and protein expression in hiPSC-CMs. (related to Figure 2 and 4)

(a) Immunoblot analysis of phospho-AKT, CDK4, and CDK1 protein levels in hiPSC-CMs treated with control, Torin1, siPRDM16, or siPRDM16 + Torin1. β-actin serves as a loading control.

(b) Quantification of Mitotracker fluorescence intensity in hiPSC-derived cardiomyocytes under control and siPRDM16 conditions, with or without Torin1 treatment. Data is shown as mean ± SD; ns, not significant, n=3 independent experiments.

(c) Representative confocal images of hiPSC-CMs stained with Mitotracker (red) and Hoechst (blue) under control, Torin1, siPRDM16, and siPRDM16 + Torin1 conditions. Scale bar, 20 μm.

(d) Immunoblot analysis of mitochondrial respiratory chain complexes (I–V) in control and siPRDM16 hiPSC-CMs, showing comparable levels across conditions. GAPDH serves as a

loading control.

Figure S4. PRDM16 knockdown does not induce structural abnormalities or DNA damage in hiPSC-derived cardiomyocytes (related to Figure 4)

(a) Representative brightfield images of three-dimensional engineered heart tissues (3D-EHTs) generated from control and siPRDM16 hiPSC-derived cardiomyocytes, showing comparable tissue morphology. Scale bar, 500 μm.

(b) RNA-seq analysis of sarcomeric and isoform markers (TNNI1, TNNI3, MYH6, MYH7, MYL7) in control and siPRDM16 hiPSC-CMs, treated with or without Torin1. Expression values are normalized to control conditions (dashed line), n=3 independent cultures per condition.

Figure S5. Lentiviral transduction efficiency and mitochondrial integrity in PRDM16 overexpression experiments (related to Figure 5)

(a) Representative phase-contrast and fluorescence images of hiPSC-derived cardiomyocytes transduced with increasing doses of GFP-expressing lentivirus (500, 1,000, 2,000, and 4,000 copies per cell), used to calibrate viral transduction efficiency. Scale bar, 200 μm.

(b) Flow cytometric analysis quantifying the percentage of GFP^+ cardiomyocytes at each viral dose shown in (a), demonstrating dose-dependent increases in transduction efficiency.

(c) Immunoblot analysis of mitochondrial respiratory chain complexes (I–V) in control and PRDM16-overexpressing hiPSC-CMs. GAPDH serves as a loading control.

Figure S6. PRDM16 overexpression suppresses cardiomyocyte cell-cycle activity (related to Figure 5)

(a) Immunofluorescence staining of hiPSC-derived cardiomyocytes for TNNI3 (red) and phospho-histone H3 (pH3) (green), with Hoechst nuclear counterstain (blue), under control and PRDM16-overexpressing conditions. Arrowheads indicate $pH3^+$ cardiomyocytes. Scale bar, 200 μm.

(b) Immunofluorescence staining of hiPSC-CMs for cardiac troponin T (TNNT2) (green) and KI-67 (red), with Hoechst nuclear counterstain (blue), under control and PRDM16-overexpressing conditions. Arrowheads mark KI-67^+ cardiomyocytes. Scale bar, 200 μm.

(c) Immunoblot analysis of cell-cycle and maturation-associated proteins in control and PRDM16-overexpressing hiPSC-CMs, including CDK1, phospho-RB1, p21, phospho-AKT, and TNNI3. β-actin serves as a loading control.

Table S1. List of qRT-PCR primers (related to Figures 1,5, S1)

**Genes**	**Sequences(5'-3')**
ACTB	**F:** CATGTACGTTGCTATCCAGGC
	**R:** CTCCTTAATGTCACGCACGAT
TNNI3	**F:** TTTGACCTTCGAGGCAAGTTT
	**R:** CCCGGTTTTCCTTCTCGGTG
PRDM16	**F:** AGATTCGGGATATGCTGTTGGC
	**R:** TCGGGTCCTAGACCAGTGTTC
MYL2	**F:** TGTCCCTACCTTGTCTGTTAGCCA
	**R:** ATTGGAACATGGCCTCTGGATGGA
IRX4	**F:** TTGGACTCCTGGGAACATGGACAA
	**R:** ATGCTTCAGGGTATCTGGCCTCTT
HEY2	**F:** AAGGCGTCGGGATCGGATAA
	**R:** AGAGCGTGTGCGTCAAAGTAG
NPPA	**F:** GGGTCTCTGCTGCATTTGTGTCAT
	**R:** AGAGGCGAGGAAGTCACCATCAAA
PITX2	**F:** GCAGCGGACTCACTTTACCA
	**R:** TAAGGTTGGTCCACACAGCG
NR2F2	**F:** TGCACGTTGACTCAGCCGAGTA
	**R:** AAGCACACTGAGACTTTTCCTGC
